# Genome-Guided Discovery of the Myxobacterial Thiolactone-Containing Sorangibactins

**DOI:** 10.1021/acschembio.3c00063

**Published:** 2023-04-04

**Authors:** Yunsheng Gao, Christine Walt, Chantal D. Bader, Rolf Müller

**Affiliations:** † Department of Microbial Natural Products, Helmholtz-Institute for Pharmaceutical Research Saarland (HIPS), Helmholtz Centre for Infection Research (HZI) and Department of Pharmacy at Saarland University, Campus E8.1, 66123 Saarbrücken, Germany; ‡ Helmholtz International Lab for Anti-Infectives, Campus E8.1, 66123 Saarbrücken, Germany; § German Center for Infection Research (DZIF), Partner Site Hannover-Braunschweig, 38124 Braunschweig, Germany

## Abstract

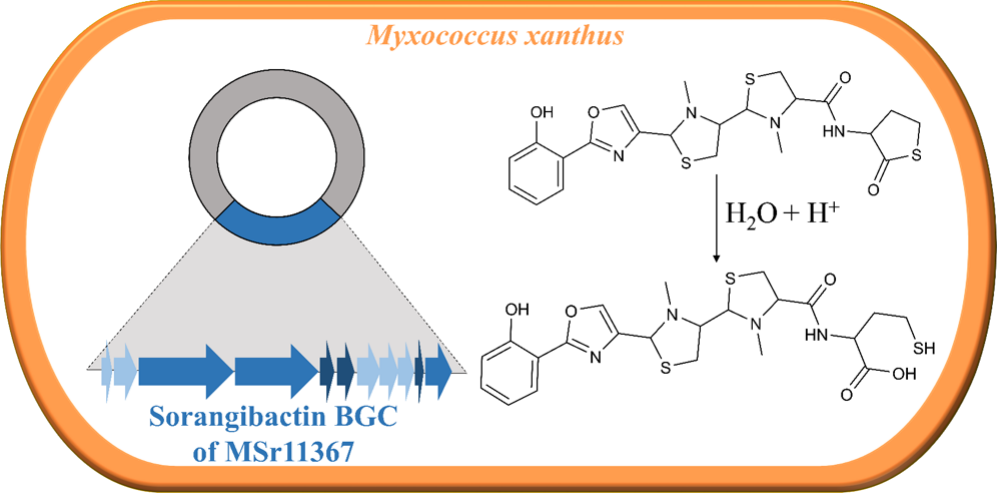

In this study, an unprecedented myxobacterial siderophore termed sorangibactin was discovered by heterologous expression of a coelibactin-like nonribosomal peptide synthetase (NRPS) gene cluster from the *Sorangiineae* strain MSr11367 in the host *Myxococcus xanthus* DK1622. De novo structure elucidation uncovered a linear polycyclic structure consisting of an N-terminal phenol group, an oxazole, tandem *N*-methyl-thiazolidines, and an unusual C-terminal γ-thiolactone moiety. Except for the unprecedented oxazoline dehydrogenation to form an oxazole, which we show to be catalyzed by a cytochrome P450-dependent enzyme, other tailoring steps were found necessary for efficient downstream processing. The unusual thioesterase (TE) domain is proposed to select homocysteine or methionine for offloading involving an intramolecular γ-thiolactone formation. Its active site comprises a rare cysteine, which was found essential for product formation by point mutation to alanine or serine, which both abolished its activity. This unusual release mechanism and the resulting rare thiolactone structure can serve as a starting point for detailed biochemical investigations.

## 1 Introduction

Myxobacteria are Gram-negative δ-proteobacteria featuring unique morphological characteristics, complex life cycles, large genomes, and a particularly huge biosynthetic potential for secondary metabolite production.^1^ Hundreds of structurally interesting scaffolds and bioactive compounds from myxobacteria were discovered over the past decades.^2^ Despite this rich collection of characterized secondary metabolites, genome sequencing as well as biosynthetic gene cluster (BGC) annotation have revealed that the majority of the myxobacterial biosynthetic potential is still untapped.^3−5^ Thus, a “gene to compound” strategy can shed light on assigning secondary metabolites to their BGCs and finding unprecedented structures. As myxobacteria, especially the *Sorangiineae* suborder, are usually slow growing and barely tractable for genetic manipulation, heterologous expression is particularly promising to explore them, as it facilitates not only genome mining for natural product discovery but also biosynthetic pathway engineering and detailed biosynthesis studies.^6^ One prolific starting point for the examination of cryptic BGCs are so-called broad cosmid libraries,^7^ which can comprise the entire genome of a selected bacterial strain in the form of plasmid DNA and facilitate high-throughput heterologous expression.^8^ This approach also enables the unambiguous correlation of BGCs with their products.

One interesting structural class of natural products are bacterial siderophores, which are small-molecular-weight Fe(III)-specific ligands that scavenge iron from their environment and enable uptake into the cell through outer-membrane transporters.^9^ Siderophores from myxobacteria belong to two main categories, namely, the catecholate-type such as the myxochelins^10^ and hyalachelins^11^ and the citrate-hydroxamate-type such as the nannochelins.^12^ On the contrary, over 500 congeners of diverse siderophore families have been described from actinobacteria.^13^ One particularly puzzling actinobacterial siderophore is coelibactin, the existence of which has only been proposed based on the presence of the respective BGC and for which only a hypothetical chemical structure is found in the literature. Comprehensive bioinformatics analysis of the respective BGC found in the model species *Streptomyces coelicolor* A3(2)^14^ enabled a prediction of the core structure based on the predicted sequence of the nonribosomal peptide synthetase (NRPS) gene cluster, yet isolation of coelibactin or derivatives thereof failed for unknown reasons.^15^

In this study, we report the discovery of a novel myxobacterial secondary metabolite with siderophore-like behavior termed sorangibactin from the *Sorangiineae* strain MSr11367 by heterologous expression of a promoter-engineered NRPS gene cluster, which resembles the coelibactin BGC. This coelibactin-like NRPS gene cluster was prioritized due to the following reasons: (1) its bioinformatically predicted structure resembles coelibactin from *Streptomyces coelicolor* A3(2),^14^ which raises hope to contribute to solving the puzzle of the coelibactins, (2) its regular operon organization, which is suitable for promoter engineering, and (3) multiple tailoring genes surrounding the core NRPS part, which indicates auxiliary modifications before or after chain release and may endow the final product with structural novelty and diversity.

## 2 Results and Discussion

### 2.1 Heterologous Expression of a Coelibactin-like NRPS Gene Cluster

**Figure 1:**
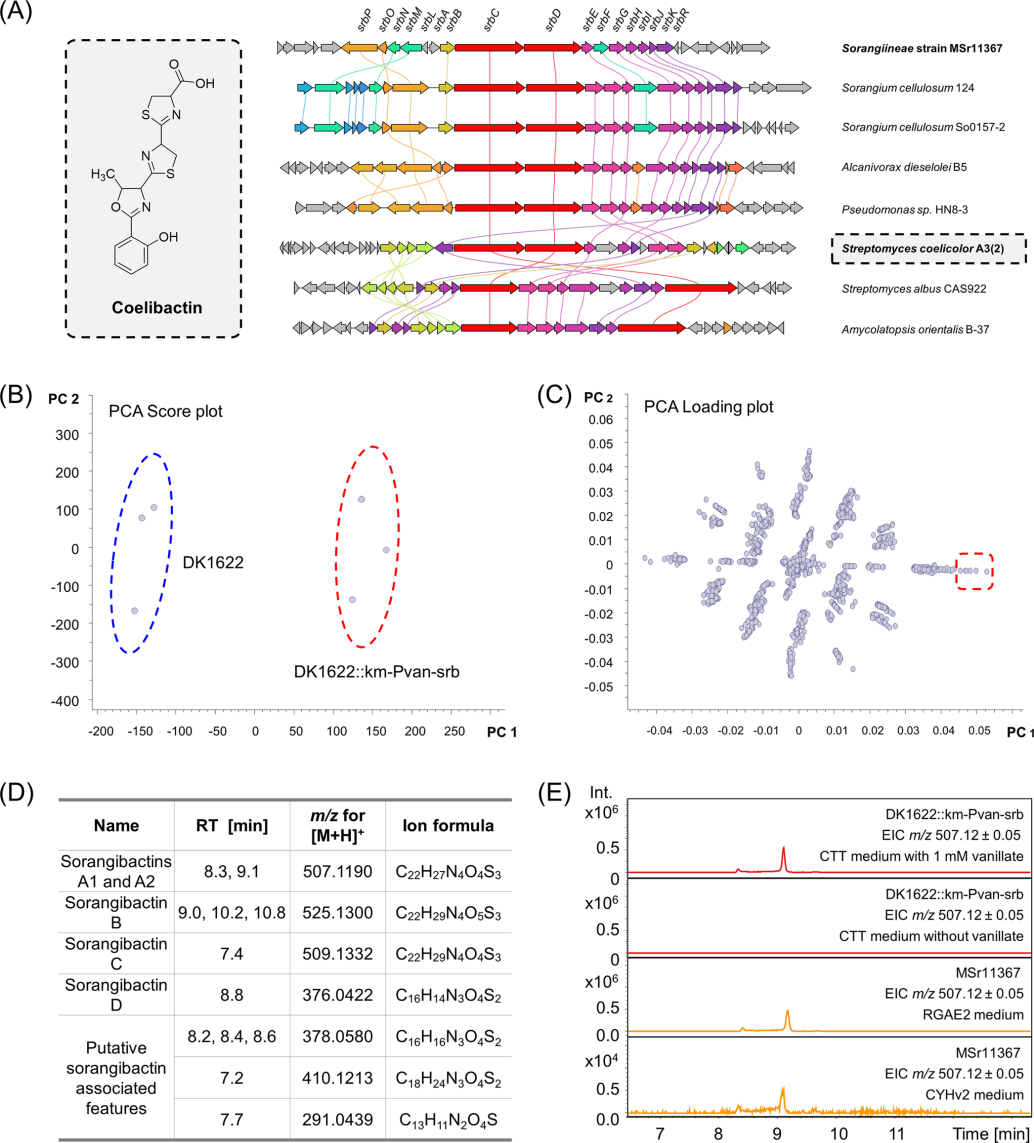
Characterization of the coelibactin-like NRPS gene cluster from the *Sorangiineae* strain MSr11367. (A) Proposed structure of coelibactin from *S. coelicolor* A3(2) and clinker analysis of selected coelibactin BGC homologues from different bacterial species. The BGC from MSr11367 with gene names is shown at the top and has been characterized in this study. Genes with a >30% sequence identity are assigned a unique color and linked. (B) Significant difference between the heterologous expression data set (DK1622::km-Pvan-srb) encircled in red and the wild-type host data set (DK1622) encircled in blue shown by a PCA score plot. (C) Molecular features that contribute to this difference shown by a PCA loading plot. (D) Molecular features that are only found in the heterologous expression data set. (E) HPLC-MS chromatogram of sorangibactin A production by DK1622::km-Pvan-srb (red) and MSr11367 (orange).

According to the antiSMASH prediction,^16^ the myxobacterial *Sorangiineae* strain MSr11367 exhibits a great biosynthetic potential of 62 biosynthetic gene clusters and thus was prioritized for natural product discovery. The strain was already explored using an OSMAC (one strain many compounds) metabolomics approach in our laboratory, but even altered cultivation conditions did not reflect its full biosynthetic potential making this strain particularly interesting for genome-based discovery. Despite several efforts, MSr11367 proved to be intractable for in situ genetic manipulation. Therefore, a genomic DNA library in the form of cosmids was generated for systematic screening of biosynthetic gene clusters by heterologous expression in the genetically amenable host *Myxococcus xanthus* DK1622.^17^ Among the 62 BGCs encoded in the strain’s genome, we prioritized a coelibactin-like NRPS gene cluster that showed widespread occurrence of homologous BGCs in *Sorangium cellulosum*, *Streptomyces*, and *Pseudomonas* strains (Figure 1A), presumably indicating a conserved function of the correlating secondary metabolites during evolution. The MSr11367 coelibactin-like gene cluster (sorangibactin BGC) has been deposited in the GenBank under the accession number OQ368584. The respective annotation of the gene cluster is provided in Supporting Table S1.

The unmodified gene cluster was cloned from the cosmid library of the *Sorangiineae* strain MSr11367 and transferred to the host *M. xanthus* DK1622 for expression; however, no heterologous product was detected. Thus, a *km-vanR-Pvan* cassette was inserted between the two main operons (Figure S1), after which the first operon consisting of transporter genes shares a strong constitutive promoter with the kanamycin resistance gene and the second operon consisting of biosynthesis genes is under the control of a vanillate-inducible Pvan promoter. Principal component analysis (PCA) of high-performance liquid chromatography–high-resolution mass spectrometry (HPLC-HRMS) datasets revealed several molecular features exclusively present in *M. xanthus* DK1622 mutants containing the promoter-engineered gene cluster and absent in the *M. xanthus* DK1622 wild-type host (Figure 1B–D). The major component (termed sorangibactin A) could also be found in *Sorangiineae* strain MSr11367 extracts in equal concentrations when cultivated under iron-deficient conditions, indicating it as the final product of the respective BGC (Figure 1E). Isolation for structure elucidation was performed utilizing the heterologous host, as besides its less complex metabolome that was beneficial for the purification process, it also shows faster growth rates reducing possible contamination issues during cultivation as observed for the strain MSr11367.

### 2.2 Characterization of Sorangibactins

**Figure 2:**
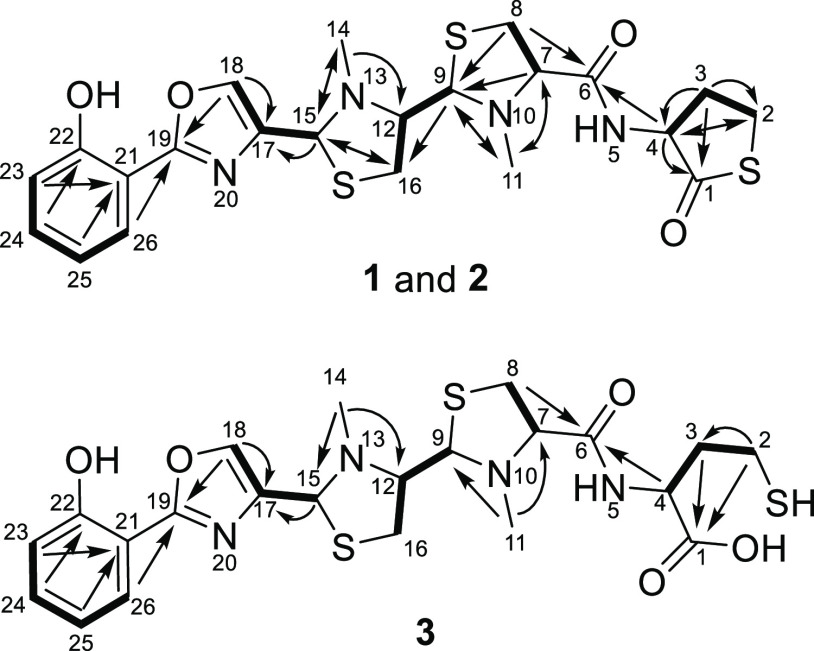
Chemical structures of sorangibactins A1 and A2 (**1** and **2**) and B (**3**) with atom numbering and main COSY (bold) and HMBC (arrows) correlations.

HPLC-HRMS measurements exhibit an [M + H]^+^ peak for the mixture of sorangibactins A1 and A2 at *m*/*z* 507.1190 matching the molecular formula C_22_H_27_N_4_O_4_S_3_^+^ (*m*/*z* calcd for [M + H]^+^ 507.1189, Δ0.2 ppm) featuring 12 double-bond equivalents (DBEs). Combined one-dimensional (1D) and two-dimensional (2D) NMR spectra uncovered a system comprising four heterocycles and one phenol, starting from the N-terminus with a phenol substituted by an oxazole group in the *ortho*-position, which is further connected to two consecutive *N*-methylated thiazolidines linked via an amide bond to a cyclic γ-thiolactone as the C-terminal end (Figure 2). The assignment of the oxazole moiety and the unusual C-terminus was underpinned by ^1^H-^15^N HMBC experiments (see the SI) as well as the comparison of the chemical shifts for the homocysteine thiolactone part to already published NPs like thiolactomide.^18^ The two signal sets for sorangibactins A1 and A2 were identical besides the signal area of the isomerization of the C-terminal homocysteine thiolactone upon purification. The corresponding methine signal for atom 4 of sorangibactin A1 is located at δ(^1^H) = 4.09 ppm and of A2 at δ(^1^H) = 4.16 ppm, whereas the adjoining diastereotopic methylene group (atom 3) of sorangibactin A1 shows a signal at δ(^1^H) = 2.10/1.92 ppm and A2 features a methylene group without any diastereotopic effect at δ(^1^H) = 2.04 ppm. It is worth noting that, in the thiolactone part, the HMBC correlation between the methylene group (atom 2) at δ(^1^H) = 3.01 ppm and the carbonyl group (atom 1) at δ(^13^C) = 179.5 ppm could not be identified. Nevertheless, the predicted thiolactone ring is underpinned by the COSY and HMBC correlations between atoms 2 and 3 as well as atom 4 by the HMBC correlations from atom 4 and atom 3 to atom 1 as well as the predicted 12 DBEs in agreement with the predicted sum formula.

Several structure parts of sorangibactin show similarities to well-described siderophores; however, it features some uncommon modifications. The *ortho*-phenol moiety originating from salicylic acid incorporation is also present in yersiniabactin produced by *Yersinia pestis*(19) and predicted for coelibactin from *Streptomyces coelicolor*.^14^ Furthermore, yersiniabactin features one thiazolidine moiety and two dihydrothiazoles, whereby the thiazolidine in contrast to sorangibactin is not *N*-methylated and no dihydrothiazoles are present in sorangibactin. The oxazole moiety in sorangibactin is different from the methylated dihydrooxazole predicted for coelibactin. Finally, the main structural difference of sorangibactin to published siderophores is clearly the uncommon homocysteine thiolactone at the C-terminus, reflecting the unprecedented structure of sorangibactin. This structural moiety impeded the purification procedure and also sheds light on possible obstacles that might have hindered structure elucidation of coelibactin: sorangibactins A1 and A2 undergo hydrolysis during the standard purification setup with formic acid as a modifier, resulting in the C-terminally ring-opened sorangibactin B. The α-proton of the acid labile homocysteine thiolactone tends to isomerize even when using the adapted purification workflow leading to a mixture of the two isomers sorangibactins A1 and A2 (see the Methods section). Even though we could not separate these two isomers, the corresponding NMR data set of the mixture enabled the structural assignment of both of them.

For the stereochemical assignment of sorangibactins A1 and A2, only Marfey’s derivatization assay was applicable since this molecule is a peptide without any functionalities accessible for Mosher esterification and due to its instabilities not attainable for crystallization experiments as exemplarily performed to solve the stereochemistry of the related siderophore yersiniabactin.^20^ Marfey’s analysis however could also only provide limited information for three main reasons: (1) the hydrolysis of thiazolidines is known to lead to the oxidation state of an aldehyde instead of the free acid, which is instable and degrades,^21^ (2) the stereocenters at positions 9 and 15 originate from the former carboxyl function of the cysteine before cyclization to the thiazolidine group and thus would lose stereo information again upon hydrolysis, and (3) the mixture of sorangibactins A1 and A2 contains both *R*- and *S*-configurations of the α-proton of the C-terminal homocysteine thiolactone, which cannot be separated. Consequently, only positions 7 and 12 could reasonably be accessible by Marfey’s analysis, but they were found to isomerize during hydrolysis. HPLC-HRMS analysis displayed a mixture of four enantiomers with a mass of *m*/*z* 430.14 ± 0.02 corresponding to FDLA-derivatized *N*-methyl cysteine and homocysteine (Figure S2). Hence, we can solely present the flat structure with a bioinformatic prediction of all *S*-configurations due to the absence of an epimerase in the respective modules.

We also obtained a second congener of the sorangibactin family through provoking hydrolysis of the rather labile thioester yielding the ring-opened product sorangibactin B (Figure 2). HPLC-HRMS experiments show an [M + H]^+^ peak for sorangibactin B at *m*/*z* 525.1298 with a predicted molecular formula C_22_H_28_N_4_O_5_S_3_^+^ (*m*/*z* calcd for [M + H]^+^ 525.1295, Δ0.6 ppm) featuring 11 double-bond equivalents (DBEs). Noteworthily, more than five isomers of sorangibactin B are present in fractions that underwent the hydrolyzing isolation conditions using formic acid during purification. The presumably isomerizing stereocenters are most likely the ones belonging to the homocysteine and the two thiazolidine parts exhibiting five stereocenters. Thus, the following structure elucidation belongs to the most abundant isomer unveiling the most intensive NMR correlations. In contrast to sorangibactins A1 and A2, the cyclic homocysteine thiolactone is opened, which shows an impact on the corresponding shifts due to the more shielded free carboxylic acid (atom 1) with a shift at δ(^13^C) = 176.6 ppm compared to the former thioester function. The HMBC spectrum exhibits two correlations to the free carboxyl group (atom 1) from methylene groups (atoms 2 and 3) at δ(^1^H) = 2.27 and 1.59 ppm. The more downfield-shifted signal can be explained by the close neighborhood to the thiol function. Corresponding COSY correlations from the more shielded methylene group (atom 3) to the homocysteine α-proton (atom 4) at δ(^1^H) = 4.68 ppm can be observed. Furthermore, HMBC correlations evince from atom 4 as well as the methine group of the adjacent thiazolidine ring (atom 7) at δ(^1^H) = 3.57 ppm to the connective amide group (atom 6) at δ(^13^C) = 173.0 ppm. Comparing the remaining data set to sorangibactins A1 and A2, the signals corresponding to the N-terminal part from atom 8 to 26 are similar with ≤0.1 ppm deviation for ^1^H and ≤2.1 ppm for ^13^C shifts.

**Figure 3:**
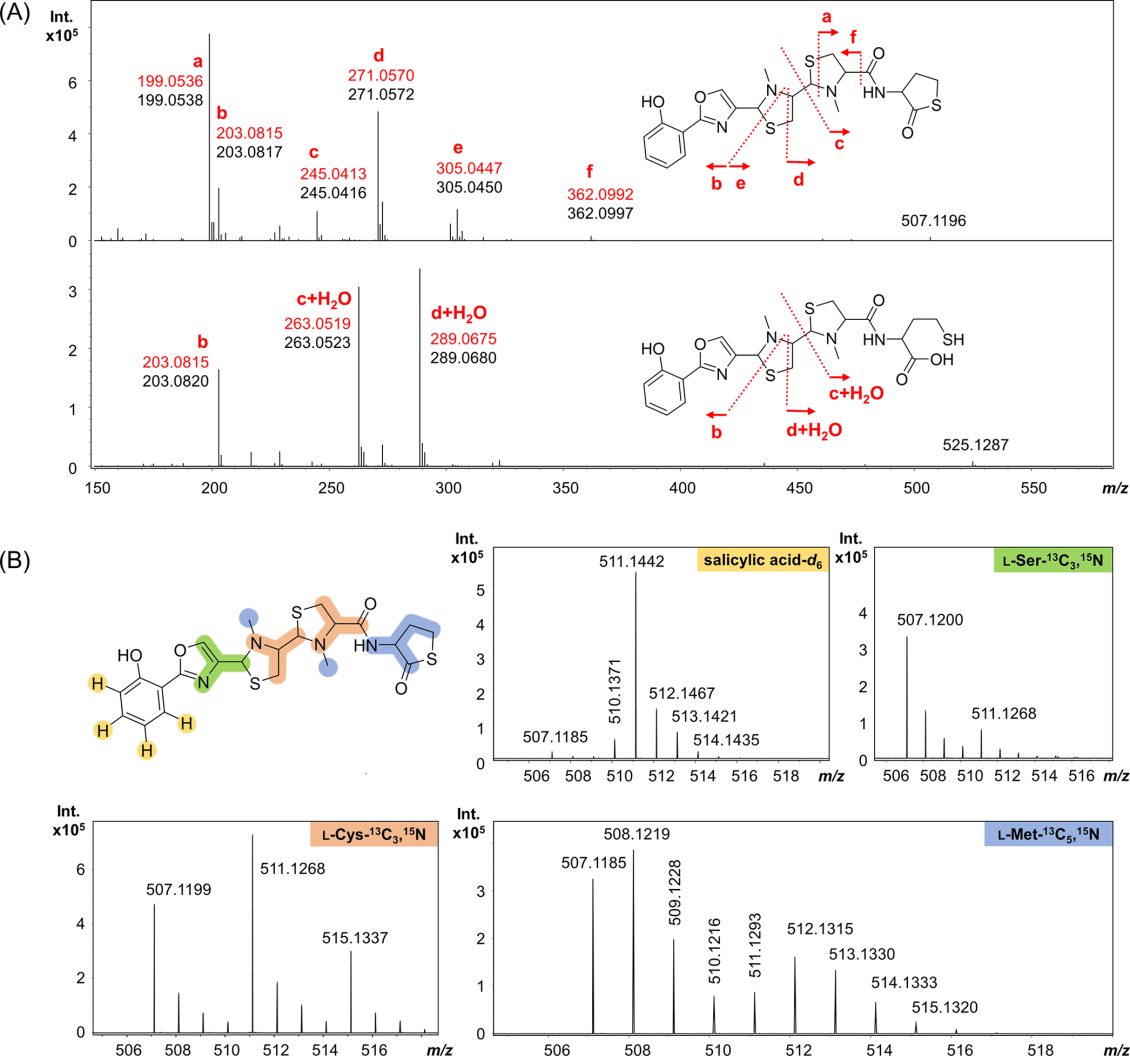
Characterization of sorangibactins by MS^2^ analysis and feeding stable isotopes. (A) Proposed fragmentation pattern of **1** and **2** with abundant fragment ions a–f (upper chromatogram) and of **3** with fragment ions b, c+H_2_O, and d+H_2_O (lower chromatogram). The predicted *m*/*z* of each fragment is marked in red. (B) Feeding salicylic acid-*d*_6_ (yellow), l-serine-^13^C_3_,^15^N (green), l-cysteine-^13^C_3_,^15^N (orange), and l-methionine-^13^C_5_,^15^N (blue).

MS^2^ spectral analysis was performed to obtain additional evidence supporting the proposed structures for sorangibactins A1, A2, and B. Just as for the structurally related siderophores yersiniabactin, ulbactin B, and escherichelin, characteristic fragmentation behavior can also be observed for sorangibactins.^22^ The three main fragment ions of sorangibactin B are in accordance with the respective bond cleavages in A1 and A2 (Figure 3A). Fragment ions b, c, and d of **1** and **2** correspond to fragment ions b, c+H_2_O, and d+H_2_O of **3**.

The N-terminal phenol group derived from salicylic acid is further supported by feeding 2-hydroxybenzoic acid-*d*_6_, resulting in a +4 mass shift of the respective fragment (Figure 3B). Feeding l-serine-^13^C_3_,^15^N and l-cysteine-^13^C_3_,^15^N resulted in the +4 and +8 mass shift, respectively, which indicates the NRPS to incorporate one serine and two cysteines and is consistent with the proposed sorangibactin structure containing one oxazole and two thiazolidine substructures. Interestingly, feeding l-methionine-^13^C_5_,^15^N not only showed a +1 mass shift indicating a methyl transfer but also a +5, which indicated the incorporated homocysteine originating from methionine at the C-terminus, which is additionally supported by MS^2^ analysis. This is consistent with the C-terminal γ-thiolactone structure.

Sorangibactins A1 and A2 showed no obvious bioactivity against our test panel of bacterial and fungal pathogens and selected cell lines (Table S2). However, siderophore-like behavior was observed during UHR-TOF measurements even before the purification of sorangibactin. Besides the double charged species at *m*/*z* 254.0632 and the [M + H]^+^ signal at *m*/*z* 507.1190, the MS spectrum of sorangibactins A1 and A2 exhibits a peak at *m*/*z* 560.0307. With a mass distance of *m*/*z* 52.9117 to the [M + H]^+^ peak, it equals [M – 2H + Fe^3+^]^+^ with the predicted sum formula of C_22_H_24_FeN_4_O_4_S_3_^+^ (*m*/*z* calcd for [M – 2H + Fe^3+^]^+^ 560.0304, Δ0.5 ppm) and shows a characteristic M-2 iron isotope peak (Figure S3).^23^ Additional evidence about sorangibactin being a siderophore is obtained by cultivation of MSr11367 under iron-deficient conditions using RGAE2 medium and iron-rich conditions by supplementing 1 mM FeCl_3_. As a result, iron limitation during cultivation induced the production of sorangibactin, whereas external addition of iron suppressed the production (Figure S4). This is a well-established indicator for siderophore features of molecules.^24,25^ Apart from that, possibilities to determine the exact affinity to iron in terms of binding properties is commonly conducted via competition experiments with a strong binder like EDTA.^26^ However, this approach was not possible for the sorangibactins due to their instability and isomerization issues during the purification process.

### 2.3 Proposed Biosynthesis of the Sorangibactins

The sorangibactin BGC spans approximately 32 kb of DNA sequence, from which 17 proteins are annotated (Table S1) and proposed to be involved in biosynthesis (SrbA–SrbK), transportation (SrbL–SrbP), and regulation (SrbR). Besides in silico analysis, gene deletion and point mutation by Redαβ recombineering (see the Methods section and Figure S1) were carried out using the heterologous expression system. Based on those experiments, we present the following biosynthesis hypothesis of the sorangibactins.

**Figure 4:**
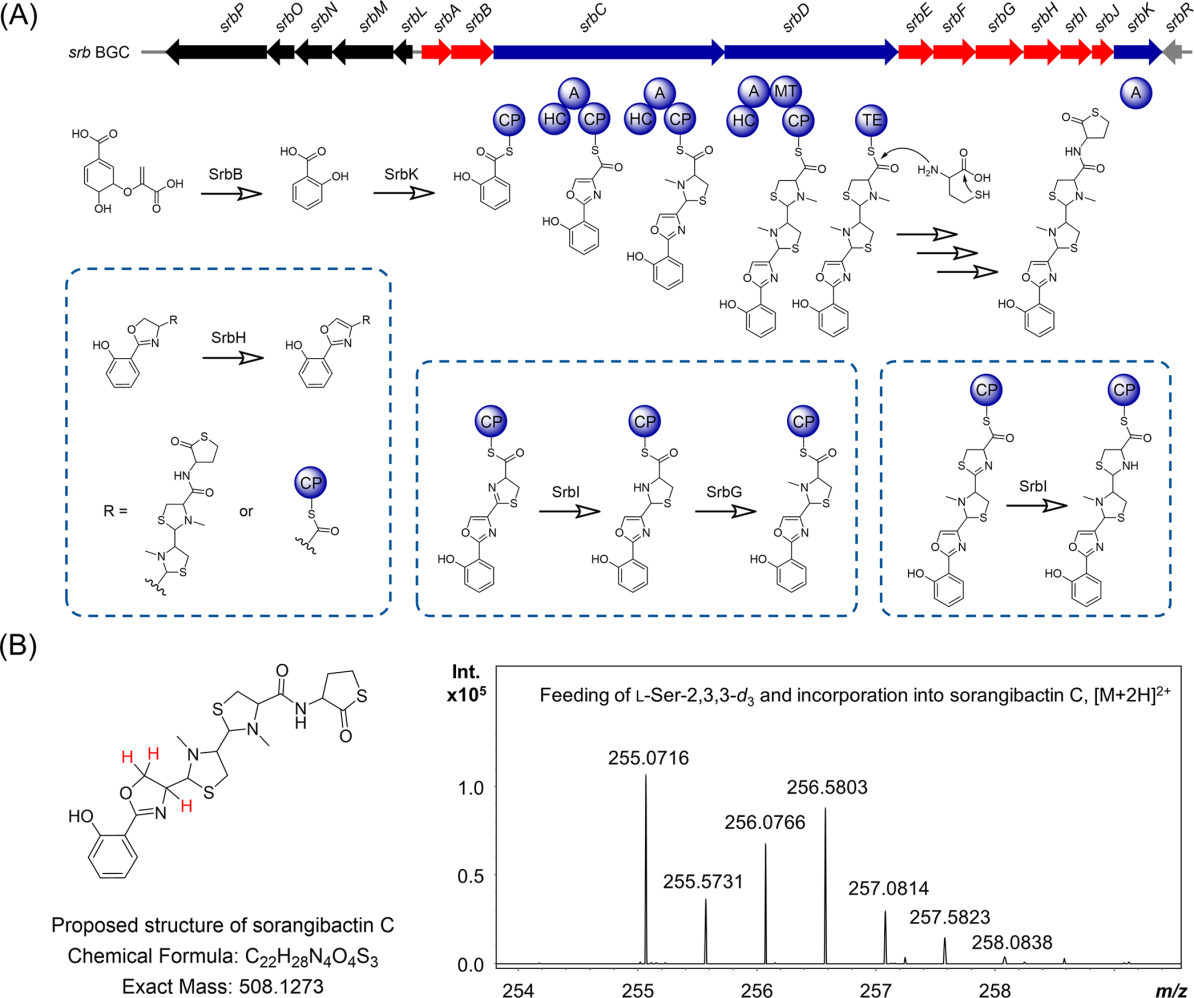
Characterization of the biosynthesis of sorangibactins. (A) Gene cluster organization and proposed biosynthetic pathway. The core biosynthetic genes are marked in blue, the proposed additional biosynthetic genes are marked in red, the proposed transport-related genes and regulatory genes are marked in black and gray, respectively. CP: carrier protein domain, HC: heterocyclization domain, A: adenylation domain, MT: methyltransferase domain, and TE: thioesterase domain. (B) Feeding of l-Ser-2,3,3-*d*_3_ and incorporation into sorangibactin C, where the proposed deuterium incorporation sites are marked in red.

Biosynthesis of the sorangibactins was initiated by salicylic acid formation catalyzed by SrbB, which shows 48.5% similarity with Irp9, a bifunctional salicylate synthase from *Yersinia enterocolitica* that can convert chorismate to salicylic acid in yersiniabactin biosynthesis.^27^ Deletion of *srbB* abolished heterologous production of sorangibactins in *M. xanthus* DK1622, whereas feeding salicylic acid to the *srbB* knockout mutant restored the production (Figure S5). The salicylic acid was then adenylated and loaded to the first carrier protein of SrbC by SrbK (Figure 4A), a stand-alone A domain-containing protein, which is predicted to use salicylic acid as a substrate (Table S3). Deletion of *srbK* completely abolished the heterologous production of sorangibactins in *M. xanthus* DK1622 (Figure S6). SrbC and SrbD are nonribosomal peptide synthases, which comprise seven and five domains, respectively (Figure 4A). Each adenylation domain was predicted by antiSMASH to use cysteine as a substrate (Table S3); however, our biosynthesis model rather suggests the first adenylation domain in SrbC to activate serine instead, which is further modified to form the oxazole incorporated in the sorangibactin structure. This is consistent with the above-mentioned result from feeding l-serine-^13^C_3_,^15^N (Figure 3B). The terminal thioesterase (TE) domain utilizes a cysteine as the active-site nucleophile instead of commonly used serine (Figure S7), which is not unprecedented but rare.^28^ Point mutation of this cysteine to alanine or serine (Figure S8) abolished the heterologous production of sorangibactin A in *M. xanthus* DK1622 (Figure S6), which indicates its essential role. Besides, this TE domain was proposed to select homocysteine or methionine as an intermolecular nucleophile for chain release resulting in one more amino acid incorporation at the C-terminus, which was confirmed by feeding l-methionine-^13^C_5_,^15^N (Figure 3B). Use of a free amino acid for chain release was previously found in threonine-tagged lipopeptide biosynthesis, where the TE domain selects the intermolecular nucleophiles threonine hydroxy or amino groups yielding ester- and amide-linked threonine tags.^29^ However, the complete C-terminal homocysteine thiolactone formation in sorangibactin biosynthesis is still unclear and may involve other tailoring enzymes, which is still under investigation.

Besides SrbK and SrbB–SrbD for the core biosynthesis, SrbE–SrbJ are proposed to be tailoring enzymes that work in trans. SrbE is a putative oxidoreductase containing a saccharopine dehydrogenase NADP-binding domain (PF03435.17) and SrbF/G are proposed to be methyltransferases. Gene deletion of SrbE/F/G was achieved by replacing them with an ampicillin resistance gene, followed by a Ptet promoter to drive the downstream expression avoiding polar effects (Figure S1), which resulted in the abolishment of sorangibactin A production and indicated their essential role for biosynthesis (Figure S6). SrbH is a cytochrome P450-dependent enzyme, sharing remarkable similarity with the CYP105N1 from the *S. coelicolor* A3(2) coelibactin biosynthetic pathway both in sequence (36.2% identity, 51.6% similarity) and structure (RMSD 1.895, Figure S9). Structure characterization of CYP105N1 revealed an unexpected wide open substrate binding pocket, which was proposed to bind the peptidyl carrier protein (PCP)-bound substrate; however, the reaction catalyzed by CYP105N1 is still unclear.^30,31^ Deletion of *srbH* abolished sorangibactin A production in *M. xanthus* DK1622, whereas another compound, termed sorangibactin C, was found accumulated (Figure S6). Molecular ion clusters [M + H]^+^ (*m*/*z* 509.1332) and [M + 2H]^2+^ (*m*/*z* 255.0716) in ESI-HRMS revealed the elemental formula C_22_H_28_N_4_O_4_S_3_ of sorangibactin C, which shows a highly similar fragmentation pattern with sorangibactin A and contains two more atoms of hydrogen in the first two cyclic moieties (Figure S10). Feeding l-serine-2,3,3-*d*_3_ revealed that these two atoms of hydrogen originated from serine, and therefore, oxazoline instead of oxazole is proposed for the sorangibactin C structure (Figure 4B). SrbH therefore presumably targets the carrier protein-bound substrate and catalyzes oxazoline dehydrogenation to form an oxazole on the assembly line (Figure 4A). However, the accumulation of sorangibactin C could also indicate SrbH to be a post-tailoring enzyme. SrbI shows 47.9% similarity with Irp3 from *Yersinia enterocolitica* (PDB: 5KVQ) and is assigned as *trans*-acting thiazolinyl imine reductase, catalyzing the NADPH-dependent reduction of a CP-tethered thiazoline ring.^32,33^ Deletion of *srbI* abolished sorangibactin A production, whereas the shunt product sorangibactin D observed at [M + H]^+^*m*/*z* 376.0422 (calculated C_16_H_14_N_3_O_4_S_2_^+^ 376.0420, Δ0.5 ppm) was accumulated (Figure S11), which indicates that the thiazoline reduction is necessary for downstream processing, otherwise resulting in early hydrolytic release. SrbJ is proposed to belong to the VOC (vicinal oxygen chelate) superfamily that catalyzes diverse reactions.^34^ The function of SrbJ in sorangibactin biosynthesis is unclear but indispensable, as of *srbJ* deletion abolished sorangibactin A production (Figure S6). At this moment, we cannot fully assign each of these biosynthesis enzymes to the structure, as the obvious accumulation of intermediate was not observed in each gene knockout, presumably due to the non-native intermediates stalling the whole machinery or chemical instabilities of the resulting shunt products.

In addition to the 10 core genes described above that were all experimentally confirmed to be directly involved in sorangibactin biosynthesis, seven additional genes were found in the sorangibactin BGC. SrbA is a proposed tailoring enzyme containing an α/β hydrolase domain. Deletion of *srbA* by Redαβ recombineering (see the Methods section and Figure S1) had no obvious influence on heterologous production of sorangibactins in *M. xanthus* DK1622 (Figures S5 and S6), which can either be explained by a functional homologue of *srbA* present in the *M. xanthus* DK1622 chromosome or *srbA* being not involved in sorangibactin biosynthesis. SrbR is an LysR family transcriptional regulator, and SrbL–SrbP are all transporter related. Whether or how the regulator and these transporters are correlated to sorangibactin production, however, was not investigated here.

## 3 Conclusions

Genome mining and subsequent heterologous expression enabled the discovery of a novel myxobacterial natural product termed sorangibactin from the myxobacterial strain MSr11367. This genomics-guided approach highlighted the accessibility of its biosynthetic potential that was not reflected in its metabolome in our previous studies. De novo structure elucidation of sorangibactin revealed the presence of an unprecedented γ-thiolactone at the C-terminus, which may help to solve the puzzle of the chemical structure of coelibactin based on their gene cluster similarity via the adapted purification route reducing putative degradation and isomerization issues as well as by providing reference data for comparison to facilitate the structure elucidation. Both biosynthetic pathways contain conserved cytochrome P450 enzymes, and the coelibactin P450 CYP105N1 protein was structurally characterized; however, their functions remained elusive before this study. Here, we discovered the sorangibactin P450-dependent enzyme SrbH, which is proposed to oxidize oxazoline to oxazole, representing the first P450-mediated oxazoline dehydrogenation in NRPS biosynthesis. Furthermore, both gene clusters involve an unusual TE domain containing a rare active-site cysteine instead of the commonly used serine. For the sorangibactin biosynthesis, we propose that homocysteine or methionine is incorporated into the C-terminus by this unusual TE domain, most probably together with other unassigned tailoring enzymes, resulting in γ-thiolactone moiety formation in a manner that is not yet fully understood. Therefore, the bioinformatically predicted coelibactin structure remains to be experimentally characterized. Future studies could address the indispensable SrbE (saccharopine dehydrogenase NADP-binding domain-containing protein, also present in the coelibactin biosynthetic pathway with yet unknown function) and SrbJ (glyoxalase-like domain-containing protein, absent in the coelibactin biosynthetic pathway but conserved in many other BGC homologues), both of which are rare in natural product biosynthesis and worthy for further biochemical investigations. Homologous gene clusters are widespread not only in myxobacteria but also in *Streptomyces* and *Pseudomonas* strains, which indicates conservative functions across bacteria and potential for further discovery and characterization of sorangibactin analogues.

In summary, this study showcased the benefit of heterologous expression with subsequent gene cluster engineering for the discovery and biosynthesis investigations of myxobacterial natural products. Accessibility of a cosmid library herein will allow further high-throughput screening of cryptic myxobacteria BGCs by heterologous expression and facilitate characterization of their yet untapped biosynthetic potential.

## 4 Methods

### 4.1 Isolation of Sorangibactins A1, A2, and B

The heterologous producer strain *M. xanthus* DK1622 mutant containing the promoter-engineered gene cluster was used for large-scale fermentation (see the SI). The myxobacterial extract in methanol was partitioned three times with hexane, dried, and redissolved in water to be partitioned three times with first ethyl acetate and later chloroform. Subsequently, the obtained chloroform phase was redissolved in methanol and fractionated with an Isolera Spektra One (Biotage) system. Respective fractions containing sorangibactins A1 and A2 were further purified using a Dionex Ultimate 3000 SL system comprising a SWPS 3000 SL autosampler, P680 pump, TCC100 column oven heated to 45 °C, PDA100 UV-detector at 220 nm, and an AFL 3000 fraction collector. In total, 2.4 mg of the mixture of sorangibactins A1 and A2 was obtained and stored under nitrogen at −20 °C until further use. For isolation of sorangibactin B, the fraction resulted from Biotage work up was treated with hydrolyzing conditions, followed by the same purification protocol compared to sorangibactins A1 and A2. Finally, 4.5 mg of sorangibactin B isomer mixture could be obtained.

### 4.2 HPLC-HRMS Measurements

All HPLC-HRMS measurements were conducted on a maXis 4G UHR-TOF system (Bruker Daltonics) equipped with an ESI source and coupled to a Dionex UltiMate 3000 rapid separation liquid chromatography (RSLC, Thermo Fisher) system. Separation was achieved with an Acquity BEH C18 column (100 mm × 2.1 mm, 1.7 μm dp) (Waters) in combination with a Waters VanGuard BEH C18 1.7 μm guard using a linear 5–95% gradient of acetonitrile with 0.1% formic acid in ddH_2_O with 0.1% formic acid for 18 min. Detection was conducted by a diode array detector at 200–600 nm, the flow rate adjusted to 0.6 mL/min, the column heated to 45 °C, and the LC flow split to 75 μL/min before entering the mass spectrometer. Following MS settings were applied for standard measurements: capillary voltage 4000 V, end plate offset −500 V, nebulizer gas pressure 1 bar, dry gas flow rate 5 L/min, dry gas temperature 200 °C, and mass scan range *m*/*z* 150–2500. MS/MS measurements were carried out in collision-induced dissociation (CID) fragmentation mode with a collision energy of 5 eV. The mass spectrometer was calibrated in quadratic + HPC calibration mode on the masses of the first isotope signal of sodium formate clusters, which are formed in the ion source after co-injection of a mixture of 1 mM sodium hydroxide in 1:1 water/isopropanol + 0.1% FA. Additional lock mass calibration was performed on *m*/*z* 622.0277, *m*/*z* 922.0103, and *m*/*z* 1221.9960.

### 4.3 Marfey’s Analysis for the Determination of Stereochemistry

For the determination of stereocenters 7 and 12 of the *N*-methylated thiazolidines as well as stereo center 4 of the cyclic homocysteine thiolactone moiety, an adapted Marfey’s derivatization protocol already described for the thiamyxins^35^ was applied after acidic hydrolysis to *N*-methyl cysteine and homocysteine, respectively.

### 4.4 NMR Measurements

For structure elucidation of sorangibactins A1 and A2, all ^1^H-, ^13^C-, and 2D-NMR spectra were acquired in methanol-*d*_4_ on a Bruker Avance III (Ascend) 700 MHz spectrometer equipped with a 5 mm TCI cryoprobe using standard pulse programs. Observed chemical shifts (δ) are listed in ppm, coupling constants (*J*) in Hz, and all spectra were calibrated with respective methanol signals at δ(^1^H) = 3.31 ppm and δ(^13^C) = 49.2 ppm. Sorangibactin B was measured using the same parameters on a Bruker Avance III (Ultrashield) 500 MHz spectrometer.

## Supporting Information Available

Materials and methods; gene cluster annotation; scheme for gene cluster engineering; domain and protein analysis; bioactivity results; and HPLC-MS and NMR data (PDF)

## References

[ref1] Gerth K.; Pradella S.; Perlova O.; Beyer S.; Müller R. Myxobacteria: proficient producers of novel natural products with various biological activities - past and future biotechnological aspects with the focus on the genus *Sorangium*. J. Biotechnol. 2003, 106, 233–253. 10.1016/j.jbiotec.2003.07.015. 14651865

[ref2] Herrmann J.; Fayad A. A.; Müller R. Natural products from myxobacteria: novel metabolites and bioactivities. Nat. Prod. Rep. 2017, 34, 135–160. 10.1039/c6np00106h. 27907217

[ref3] Bauman K. D.; Butler K. S.; Moore B. S.; Chekan J. R. Genome mining methods to discover bioactive natural products. Nat. Prod. Rep. 2021, 38, 2100–2129. 10.1039/D1NP00032B. 34734626 PMC8597713

[ref4] Panter F.; Krug D.; Baumann S.; Müller R. Self-resistance guided genome mining uncovers new topoisomerase inhibitors from myxobacteria. Chem. Sci. 2018, 9, 4898–4908. 10.1039/C8SC01325J. 29910943 PMC5982219

[ref5] Gavriilidou A.; Kautsar S. A.; Zaburannyi N.; Krug D.; Müller R.; Medema M. H.; Ziemert N. Compendium of specialized metabolite biosynthetic diversity encoded in bacterial genomes. Nat. Microbiol. 2022, 7, 726–735. 10.1038/s41564-022-01110-2. 35505244

[ref6] Huo L.; Hug J. J.; Fu C.; Bian X.; Zhang Y.; Müller R. Heterologous expression of bacterial natural product biosynthetic pathways. Nat. Prod. Rep. 2019, 36, 1412–1436. 10.1039/c8np00091c. 30620035

[ref7] Clos J.; Zander-Dinse D. Cosmid Library Construction and Functional Cloning. Methods Mol. Biol. 2019, 1971, 123–140. 10.1007/978-1-4939-9210-2_6. 30980301

[ref8] Wenzel S. C.; Müller R. Recent developments towards the heterologous expression of complex bacterial natural product biosynthetic pathways. Curr. Opin. Biotechnol. 2005, 16, 594–606. 10.1016/j.copbio.2005.10.001. 16226455

[ref9] Hider R. C.; Kong X. Chemistry and biology of siderophores. Nat. Prod. Rep. 2010, 27, 637–657. 10.1039/B906679A. 20376388

[ref10] Kunze B.; Bedorf N.; Kohl W.; Höfle G.; Reichenbach H. Myxochelin A, a new iron-chelating compound from *Angiococcus disciformis* (Myxobacterales). Production, isolation, physico-chemical and biological properties. J. Antibiot. 1989, 42, 14–17. 10.7164/antibiotics.42.14. 2493439

[ref11] Nadmid S.; Plaza A.; Lauro G.; Garcia R.; Bifulco G.; Müller R. Hyalachelins A-C, unusual siderophores isolated from the terrestrial myxobacterium *Hyalangium minutum*. Org. Lett. 2014, 16, 4130–4133. 10.1021/ol501826a. 25058569

[ref12] Kunze B.; Trowitzsch-Kienast W.; Höfle G.; Reichenbach H. Nannochelins A, B and C, new iron-chelating compounds from *Nannocystis exedens* (myxobacteria). Production, isolation, physico-chemical and biological properties. J. Antibiot. 1992, 45, 147–150. 10.7164/antibiotics.45.147. 1556005

[ref13] Wang W.; Qiu Z.; Tan H.; Cao L. Siderophore production by actinobacteria. BioMetals 2014, 27, 623–631. 10.1007/s10534-014-9739-2. 24770987

[ref14] Bentley S. D.; Chater K. F.; Cerdeño-Tárraga A.-M.; Challis G. L.; Thomson N. R.; James K. D.; Harris D. E.; Quail M. A.; Kieser H.; Harper D.; Bateman A.; Brown S.; Chandra G.; Chen C. W.; Collins M.; Cronin A.; Fraser A.; Goble A.; Hidalgo J.; Hornsby T.; Howarth S.; Huang C.-H.; Kieser T.; Larke L.; Murphy L.; Oliver K.; O’Neil S.; Rabbinowitsch E.; Rajandream M.-A.; Rutherford K.; Rutter S.; Seeger K.; Saunders D.; Sharp S.; Squares R.; Squares S.; Taylor K.; Warren T.; Wietzorrek A.; Woodward J.; Barrell B. G.; Parkhill J.; Hopwood D. A. Complete genome sequence of the model actinomycete *Streptomyces coelicolor* A3(2). Nature 2002, 417, 141–147. 10.1038/417141a. 12000953

[ref15] Johnstone T. C.; Nolan E. M. Beyond iron: non-classical biological functions of bacterial siderophores. Dalton Trans. 2015, 44, 6320–6339. 10.1039/C4DT03559C. 25764171 PMC4375017

[ref16] Blin K.; Shaw S.; Kloosterman A. M.; Charlop-Powers Z.; van Wezel G. P.; Medema M. H.; Weber T. antiSMASH 6.0: improving cluster detection and comparison capabilities. Nucleic Acids Res. 2021, 49, W29–W35. 10.1093/nar/gkab335. 33978755 PMC8262755

[ref17] Yang Y.-J.; Singh R. P.; Lan X.; Zhang C.-S.; Li Y.-Z.; Li Y.-Q.; Sheng D.-H. Genome Editing in Model Strain *Myxococcus xanthus* DK1622 by a Site-Specific Cre/loxP Recombination System. Biomolecules 2018, 8, 137 10.3390/biom8040137. 30404219 PMC6316027

[ref18] Jang J.-P.; Kwon M. C.; Nogawa T.; Takahashi S.; Osada H.; Ahn J. S.; Ko S.-K.; Jang J.-H. Thiolactomide: A New Homocysteine Thiolactone Derivative from *Streptomyces* sp. with Neuroprotective Activity. J. Microbiol. Biotechnol. 2021, 31, 1667–1671. 10.4014/jmb.2108.08015. 34528916 PMC9706031

[ref19] Haag H.; Hantke K.; Drechsel H.; Stojiljkovic I.; Jung G.; Zähner H. Purification of yersiniabactin: a siderophore and possible virulence factor of *Yersinia enterocolitica*. Microbiology 1993, 139, 2159–2165. 10.1099/00221287-139-9-2159. 8245841

[ref20] Miller M. C.; Parkin S.; Fetherston J. D.; Perry R. D.; DeMoll E. Crystal structure of ferric-yersiniabactin, a virulence factor of *Yersinia pestis*. J. Inorg. Biochem. 2006, 100, 1495–1500. 10.1016/j.jinorgbio.2006.04.007. 16806483

[ref21] Fife T. H.; Natarajan R.; Shen C. C.; Bembi R. Mechanism of thiazolidine hydrolysis. Ring opening and hydrolysis of 1,3-thiazolidine derivatives of *p*-(dimethylamino)cinnamaldehyde. J. Am. Chem. Soc. 1991, 113, 3071–3079. 10.1021/ja00008a041.

[ref22] Schulz M.; Gaitanoglou V.; Mantel O.; Hövelmann Y.; Hübner F.; Dobrindt U.; Humpf H.-U. Metabolomics Study on Pathogenic and Non-pathogenic *E. coli* with Closely Related Genomes with a Focus on Yersiniabactin and Its Known and Novel Derivatives. Metabolites 2020, 10, 221 10.3390/metabo10060221. 32481767 PMC7344775

[ref23] Hermenau R.; Mehl J. L.; Ishida K.; Dose B.; Pidot S. J.; Stinear T. P.; Hertweck C. Genomics-Driven Discovery of NO-Donating Diazeniumdiolate Siderophores in Diverse Plant-Associated Bacteria. Angew. Chem., Int. Ed. 2019, 58, 13024–13029. 10.1002/anie.201906326. PMC6771848 31276269

[ref24] Neilands J. B. Siderophores: structure and function of microbial iron transport compounds. J. Biol. Chem. 1995, 270, 26723–26726. 10.1074/jbc.270.45.26723. 7592901

[ref25] Wilhelm S. W.; Trick C. G. Iron-limited growth of cyanobacteria: Multiple siderophore production is a common response. Limnol. Oceanogr. 1994, 39, 1979–1984. 10.4319/lo.1994.39.8.1979.

[ref26] Perry R. D.; Balbo P. B.; Jones H. A.; Fetherston J. D.; DeMoll E. Yersiniabactin from *Yersinia pestis*: biochemical characterization of the siderophore and its role in iron transport and regulation. Microbiology 1999, 145, 1181–1190. 10.1099/13500872-145-5-1181. 10376834

[ref27] Kerbarh O.; Ciulli A.; Howard N. I.; Abell C. Salicylate biosynthesis: Overexpression, purification, and characterization of Irp9, a bifunctional salicylate synthase from *Yersinia enterocolitica*. J. Bacteriol. 2005, 187, 5061–5066. 10.1128/JB.187.15.5061–5066.2005. 16030197 PMC1196042

[ref28] Little R. F.; Hertweck C. Chain release mechanisms in polyketide and non-ribosomal peptide biosynthesis. Nat. Prod. Rep. 2022, 39, 163–205. 10.1039/d1np00035g. 34622896

[ref29] Thongkongkaew T.; Ding W.; Bratovanov E.; Oueis E.; Garcı A-Altares M. A.; Zaburannyi N.; Harmrolfs K.; Zhang Y.; Scherlach K.; Müller R.; Hertweck C. Two Types of Threonine-Tagged Lipopeptides Synergize in Host Colonization by Pathogenic *Burkholderia* Species. ACS Chem. Biol. 2018, 13, 1370–1379. 10.1021/acschembio.8b00221. 29669203

[ref30] Lim Y.-R.; Hong M.-K.; Kim J.-K.; Doan T. T. N.; Kim D.-H.; Yun C.-H.; Chun Y.-J.; Kang L.-W.; Kim D. Crystal structure of cytochrome P450 CYP105N1 from *Streptomyces coelicolor*, an oxidase in the coelibactin siderophore biosynthetic pathway. Arch. Biochem. Biophys 2012, 528, 111–117. 10.1016/j.abb.2012.09.001. 23000034

[ref31] Zhao B.; Moody S. C.; Hider R. C.; Lei L.; Kelly S. L.; Waterman M. R.; Lamb D. C. Structural analysis of cytochrome P450 105N1 involved in the biosynthesis of the zincophore, coelibactin. Int. J. Mol. Sci. 2012, 13, 8500–8513. 10.3390/ijms13078500. 22942716 PMC3430247

[ref32] Meneely K. M.; Lamb A. L. Two structures of a thiazolinyl imine reductase from *Yersinia enterocolitica* provide insight into catalysis and binding to the nonribosomal peptide synthetase module of HMWP1. Biochemistry 2012, 51, 9002–9013. 10.1021/bi3011016. 23066849 PMC3491095

[ref33] Meneely K. M.; Ronnebaum T. A.; Riley A. P.; Prisinzano T. E.; Lamb A. L. Holo Structure and Steady State Kinetics of the Thiazolinyl Imine Reductases for Siderophore Biosynthesis. Biochemistry 2016, 55, 5423–5433. 10.1021/acs.biochem.6b00735. 27601130 PMC5046821

[ref34] He P.; Moran G. R. Structural and mechanistic comparisons of the metal-binding members of the vicinal oxygen chelate (VOC) superfamily. J. Inorg. Biochem. 2011, 105, 1259–1272. 10.1016/j.jinorgbio.2011.06.006. 21820381

[ref35] Haack P. A.; Harmrolfs K.; Bader C. D.; Garcia R.; Gunesch A. P.; Haid S.; Popoff A.; Voltz A.; Kim H.; Bartenschlager R.; Pietschmann T.; Müller R. Thiamyxins: Structure and Biosynthesis of Myxobacterial RNA-Virus Inhibitors. Angew. Chem., Int. Ed. 2022, 61, e202212946 10.1002/anie.202212946. PMC10100342 36208117

