# Supplementary material for: Genome-Guided Discovery of the Myxobacterial Thiolactone-Containing Sorangibactins
Source: ACS Chem Biol. 2023 Apr 4;18(4):924–32. doi: 10.1021/acschembio.3c00063 (PMC10127202; doi:10.1021/acschembio.3c00063)
Supplement: Supplementary file 1 — cb3c00063_si_001.pdf [file cb3c00063_si_001.pdf]

Supporting Information

Genome-Guided Discovery of the Myxobacterial Thiolactone-Containing Sorangibactins

Yunsheng Gao[a,b,c,+], Christine Walt[a,c,+], Chantal D. Bader[a,c], Rolf Müller[a,b,c,*]

[a] Department of Microbial Natural Products, Helmholtz-Institute for Pharmaceutical Research Saarland (HIPS), Helmholtz Centre for Infection Research (HZI) and Department of Pharmacy at Saarland University, Campus E8.1, 66123 Saarbrücken, Germany

[b] Helmholtz International Lab for Anti-Infectives, Campus E8.1, 66123 Saarbrücken, Germany

[c] German Center for Infection Research (DZIF), Partner Site Hannover-Braunschweig, 38124 Braunschweig, Germany

[+] These authors contributed equally to this work

* To whom correspondence should be addressed: rolf.mueller@helmholtz-hips.de

Table of Contents

1. Methods S4

1.1 Cultivation conditions for bacterial strains S4

1.2 Gene cluster cloning and engineering S4

1.3 Heterologous expression in *M. xanthus* DK1622 S5

1.4 Comparative data analysis S5

1.5 Feeding stable isotopes S6

1.6 Fermentation and compounds purification S6

1.7 Structure elucidation S7

1.8 Marfey's analysis for determination of stereochemistry S9

1.9 Bioactivity evaluation of sorangibactin A S9

2. Supplementary tables S11

Table S1 Annotation of the sorangibactin biosynthetic gene cluster. S11

Table S2 Bioactivity results of sorangibactin A (**1** and **2**). S12

Table S3 Substrate specificities of adenylation domains in *srb* gene cluster. S12

Table S4 Oligo nucleotides used in this study. S13

Table S5 NMR spectroscopic data of sorangibactin A1 (**1**) in methanol-d_4 at 700/175 MHz. S14

Table S6 NMR spectroscopic data of sorangibactin A2 (**2**) in methanol-d_4 at 700/175 MHz. S15

Table S7 NMR spectroscopic data of sorangibactin B (**3**) in methanol-d_4 at 500/125 MHz. S16

3. Supplementary figures S17

Figure S1 Expression constructs used in this study. S17

Figure S2 HPLC-MS measurements of Marfey's derivatization of sorangibactin A (**1** and **2**). S18

Figure S3 HRMS spectrum of sorangibactin A. S18

Figure S4 Production of sorangibactin A by MSr11367 with and without iron addition. S19

Figure S5 HPLC-MS measurements of *srbA*, *srbAB* knockout mutants and feeding salicylic acid to *srbAB* knockout mutant. S19

Figure S6 HPLC-MS analysis of crude extrudes from *M. xanthus* DK1622 and mutants containing the corresponding sorangibactin expression constructs. S20

Figure S7 Alignment of TE domains. S21

Figure S8 Point mutation of C1586A and C1586S of the sorangibactin TE domain. S21

Figure S9 Superimposition of SrbH and CYP105N1. S21

Figure S10 HRMS^2 of sorangibactin C in comparison with sorangibactin A. S22

Figure S11 Proposed structure and incorporation of L-Cys-$^{13}C_3$,^{15}N of sorangibactin D. S22

Figure S12 ^{1}H-spectrum of sorangibactin A1 (**1**) in methanol-d_4 at 700 MHz. S23

Figure S13 ^{13}C-spectrum of sorangibactin A1 (**1**) in methanol-d_4 at 175 MHz. S24

Figure S14 COSY spectrum of sorangibactin A1 (**1**) in methanol-d_4 at 700 MHz. S25

Figure S15 HSQC spectrum of sorangibactin A1 (**1**) in methanol-d_4 at 700 MHz (^{1}H)/175 MHz (^{13}C). S26

Figure S16 HMBC spectrum of sorangibactin A1 (**1**) in methanol-d_4 at 700 MHz (^{1}H)/175 MHz (^{13}C). S27

Figure S17 ^{1}H-^{15}N-HMBC spectrum of sorangibactin A1 (**1**) in methanol-d_4 at 500 MHz (^{1}H)/125 MHz (^{15}N). S28

Figure S18 ^{1}H-spectrum of sorangibactin A2 (**2**) in methanol-d_4 at 700 MHz. S29

Signals corresponding to sorangibactin A1 (**1**) are highlighted in gray. S29

Figure S19 ^{13}C-spectrum of sorangibactin A2 (**2**) in methanol-d_4 at 175 MHz. S30

Figure S20 COSY spectrum of sorangibactin A2 (**2**) in methanol-d_4 at 700 MHz. S31

Figure S21 HSQC spectrum of sorangibactin A2 (**2**) in methanol-d_4 at 700 MHz (^{1}H)/175 MHz (^{13}C). S32

Figure S22 HMBC spectrum of sorangibactin A2 (**2**) in methanol-d_4 at 700 MHz (^{1}H)/175 MHz (^{13}C). S33

Figure S23 ^{1}H-^{15}N-HMBC spectrum of sorangibactin A2 (**2**) in methanol-d_4 at 500 MHz (^{1}H)/125 MHz (^{15}N). S34

Figure S24 ^{1}H-spectrum of sorangibactin B (**3**) in methanol-d_4 at 500 MHz. S35

Figure S25 ^{13}C-spectrum of sorangibactin B (**3**) in methanol-d_4 at 125 MHz. S36

Figure S26 COSY spectrum of sorangibactin B (**3**) in methanol-d_4 at 500 MHz. S37

Figure S27 HSQC spectrum of sorangibactin B (**3**) in methanol-d_4 at 500 MHz (^{1}H)/125 MHz (^{13}C). S38

Figure S28 HMBC spectrum of sorangibactin B (**3**) in methanol-d_4 at 500 MHz (^{1}H)/125 MHz (^{13}C). S39

4. References S40

1. Methods

1.1 Cultivation conditions for bacterial strains

The myxobacterial *Sorangiineae* strain MSr11367 was cultivated in 100 mL RGAE2 medium (3.5 g/L casitone, 1 g/L soytone, 0.5 g/L yeast extract, 2 g/L glucose, 2 g/L soluble starch, 1 g/L sucrose, 1 g/L maltose monohydrate, 2 g/L cellobiose, 0.5 g/L calcium chloride dihydrate, 1 g/L magnesium sulfate heptahydrate, 2.38 g/L HEPES, pH=7.0) or CYHv2 medium (4 g/L soya meal starch, 3 g/L casitone, 2 g/L glucose, 8 g/L soluble starch, 1.5 g/L yeast extract, 1 g/L calcium chloride dihydrate, 1 g/L magnesium sulfate heptahydrate, 11.9 g/L HEPES, 8 mg/L Fe-EDTA, pH=7.2) in a 300 mL flask with shaking at 180 rpm and 30 °C for 14 days. *Myxococcus xanthus* DK1622 was used as a heterologous host for gene cluster expression and cultivated in CTT medium (10 g/L casitone, 10 mM Tris-HCl, 8 mM magnesium sulfate, 1 mM potassium phosphate, pH=7.6) at 30 °C. *E. coli* strains GB2005, GB05-red, GBred-gyrA462 were used for gene cluster engineering and cultivated in LB medium (10 g/L tryptone, 5 g/L yeast extract, 5 g/L sodium chloride, pH=7.0) at 37 °C. The following antibiotics were supplemented to the medium when the cultivated strain carries the corresponding resistance gene: 10 µg/mL for oxytetracycline, 15 µg/mL for chloramphenicol, 50 µg/mL for kanamycin and 100 µg/mL for ampicillin.

1.2 Gene cluster cloning and engineering

The cosmid library for *Sorangiineae* strain MSr11367 was constructed by Explogen LLC (EXG) (https://explogen.com.ua) using genomic DNA and p15A-cm-tet-int vector. After end-sequencing, the cosmid pCos-D05_9223 was found to be able to cover the putative whole gene cluster and used for heterologous expression. For generation of promoter engineered expression construct (pCos-D05_9223-km-Pvan), a *km-vanR-Pvan* cassette amplified by PCR was electroporated into *E. coli* GB05-red harboring plasmid pCos-D05_9223 and expressing Redαβ recombinases for linear-circular homologous recombination (LCHR). Gene knockout constructs (pCos-D05_9223-km-Pvan-delA and pCos-D05_9223-km-Pvan-delAB) were generated in a similar manner except reverse oligo nucleotides containing different downstream homology arms were used. Deletion of *srbE/F/G/H/I/J/K* was based on pCos-D05_9223-km-Pvan. To do this, *amp-Ptet* cassettes amplified by PCR using corresponding oligo nucleotides (Table S4) were used to replace the target gene using LCHR. As a result, the downstream genes will be under control of a Ptet promoter in order to avoid potential polar

effect. For point mutation of the TE domain, RedEx method was used,(*1*) *amp-ccdB* cassettes containing homology arms, mutation sites and PmeI sites ware amplified by PCR using corresponding oligo nucleotides (Table S4) and electroporated into *E. coli* GBred-gyrA462 harboring plasmid pCos-D05_9223-km-Pvan for LCHR. The resulting plasmids were digested by PmeI, recircularized by Gibson Assembly and electroporated into *E. coli* GB2005. Correct recombinants were confirmed by restriction digestions and Sanger sequencing.

1.3 Heterologous expression in *M. xanthus* DK1622

Expression constructs containing the sorangibactin BGC were transferred to the heterologous host *M. xanthus* DK1622 by electroporation according to a previously established procedure.(*2*) The gene cluster was then integrated into the *attB* locus on the *M. xanthus* DK1622 chromosome through phage Mx8 integrase mediated site-specific recombination.(*3*) Then, the mutants were checked by colony PCR using three pairs of primers (Table S4) targeting different positions of the gene cluster to ensure the intact integration. Three independent mutants were inoculated in CTT medium supplemented with appropriate antibiotics and cultivated at 30 °C with shaking until late log-phase. The culture was inoculated 1 to 50 into fresh CTT medium supplemented with 2% XAD-16 resin, appropriate antibiotics and inducer (1 mM vanillate for Pvan promoter and 0.5 µg/mL anhydrotetracycline for Ptet promoter). Cultivation was continued at 30 °C with shaking for 3 days. For comparative analysis, triplicate cultivation of *M. xanthus* DK1622 wild-type strains ware performed in a similar manner except that no antibiotics were added. The cells and adsorber resin were collected by centrifugation and extracted with methanol. The crude extracts were dried by rotary evaporation *in vacuo* and redissolved in 1 mL methanol for HPLC-HRMS measurement.

1.4 Comparative data analysis

Data processing was performed using Bruker DataAnalysis 4.4 and MetaboScape 2021b. For comparative analysis, bucket tables consisting of molecular features (RT-*m/z* pairs) and their corresponding intensity values were generated using the T-ReX 3D algorithm. Principal component analysis (PCA) and t-test were used to find out unique features that are only present in the heterologous expression group, but not in the control group. These unique features were double-checked manually by extracting the corresponding ion chromatograms.

1.5 Feeding stable isotopes

The stable isotopes were prepared as stock solutions of 0.2 M and supplemented to the culture at the final concentration of 0.2 mM. To prepare stock solutions, L-cysteine-$^{13}C_3$,^{15}N (Sigma Aldrich, 658057), L-serine-$^{13}C_3$,^{15}N (Sigma Aldrich, 608130), L-serine-2,3,3-d_3 (Cambridge Isotope Laboratories, DLM-582-0.5), L-methionine-d_3 (methyl-d_3) (Campro Scientific GmbH, CS01-182_581) and L-methionine-$^{13}C_5$,^{15}N (Campro Scientific GmbH, CS01-181_212) were dissolved in autoclaved Milli-Q water, 2-hydroxybenzoic acid-d_6 (Campro Scientific GmbH, CS01-182_507) was dissolved in ethanol. The heterologous host *M. xanthus* DK1622::km-Pvan-srb was inoculated in 20 mL CTT medium supplemented with kanamycin at 30 °C with shaking until mid log-phase. After addition of 1 mM vanillate, 2% XAD-16 resin and corresponding stable isotope, cultivation was continued for two more days. The cells and adsorber resin were collected and extracted with methanol. The crude extracts were dried by rotary evaporation *in vacuo* and redissolved in 1 mL methanol for HPLC-HRMS measurement.

1.6 Fermentation and compounds purification

The heterologous producer strain *M. xanthus* DK1622::km-Pvan-srb was inoculated in CTT medium supplemented kanamycin and cultivated at 30 °C with shaking until late log-phase. The culture was inoculated 1 to 50 into 6 × 2 L fresh CTT medium supplemented with 2% XAD-16 resin, 50 µg/mL kanamycin and 1 mM vanillate in 6 × 5 L flaskes. Cultivation was continued at 30 °C with shaking for 4 days. The cells and adsorber resin were collected by centrifugation and extracted stepwise with methanol. The methanol extract was concentrated and partitioned with hexane to remove nonpolar impurity. After evaporation *in vacuo*, the methanol phase was then dissolved in Milli-Q water and extracted twice with equal portions of chloroform. The chloroform phase was evaporated to dryness and redissolved in methanol.

Isolera™ Spektra One (Biotage) system: Normal phase chromatography was performed using silica gel (60 Å, 70-230 mesh, 63-200 µm) packed in a SNAP 50g column as stationary phase and a multistep gradient with hexane, ethyl acetate and methanol as mobile phase using a 15 column volume (CV) gradients and 5 CV for equilibration at a flowrate of 50 mL/min.

Semi-preparative purification of sorangibactin A1 and A2: Separation was reached using a Waters XBridge Peptide BEH C18 column (250 × 10 mm, 5 µm) in combination with a mobile phase consisting of A = 0.1% acetic acid in ddH_2O and B = 0.1% acetic acid in acetonitrile

running a 5-95% B gradient in 16 min with a 4 min equilibration at 5% B with a flow rate of 5 mL/min. The acid labile sorangibactin A1 and A2 were pooled in a flask filled with $NaHCO_3$ for neutralization purpose, which was subsequently disjoint through liquid-liquid partitioning three times using chloroform.

Semi-preparative purification of sorangibactin B: Separation was achieved via a Waters XBridge Peptide BEH C18 column (250 × 10 mm, 5 µm) using formic acid instead of acetic acid and following adapted multi-step gradient: hold at 5% B for 1 min, increasing to 20% B within 0.5 min, 1.5-12.5 min increasing to 80% B, increasing to 95% B within 0.5 min, hold at 95% B for 1.5 min, go back to 5% B within 0.5 min and re-equilibrate for 5 min at a flow rate of 5 mL/min. The obtained fraction was kept at room temperature overnight to achieve conversion to sorangibactin B.

1.7 Structure elucidation

Structure elucidation of sorangibactin A1 and A2 mixture: ^{1}H-NMR, ^{13}C and HSQC spectra show five aromatic double-bond signals at $\delta(^{1}H)$ = 7.89 (1H, s) $\delta(^{13}C)$ = 136.4, $\delta(^{1}H)$ = 7.82 (1H, d, J = 7.80 Hz) $\delta(^{13}C)$ = 127.2, $\delta(^{1}H)$ = 7.36 (1H, m) $\delta(^{13}C)$ = 133.7, $\delta(^{1}H)$ = 7.00 (1H, d, J = 8.38Hz) $\delta(^{13}C)$ = 118.2 and $\delta(^{1}H)$ = 6.95 (1H, m) $\delta(^{13}C)$ = 120.7 ppm. Furthermore, five methine signals can be found at $\delta(^{1}H)$ = 5.01 (1H, s) $\delta(^{13}C)$ = 70.1, $\delta(^{1}H)$ = 4.21 (1H, m) $\delta(^{13}C)$ = 76.1, $\delta(^{1}H)$ = 4.09 (1H, dd, J = 6.94, 3.66 Hz) $\delta(^{13}C)$ = 63.1, $\delta(^{1}H)$ = 3.59 (1H, t, J = 7.95Hz) $\delta(^{13}C)$ = 81.2 as well as $\delta(^{1}H)$ = 3.29 (1H, m) $\delta(^{13}C)$ = 75.6 ppm. Additionally, two methyl signals are located at $\delta(^{1}H)$ = 2.57 (3H, s) $\delta(^{13}C)$ = 41.5 and $\delta(^{1}H)$ = 2.47 (3H, s) $\delta(^{13}C)$ = 40.8, one methylene signal at $\delta(^{1}H)$ = 3.01 (2H, m) $\delta(^{13}C)$ = 24.9 and three diastereotopic methylene signals at $\delta(^{1}H)$ = 3.15, 3.02 (2H, m) $\delta(^{13}C)$ = 35.3, $\delta(^{1}H)$ = 3.29, 3.16 (2H, m) $\delta(^{13}C)$ = 33.8 and $\delta(^{1}H)$ = 2.10, 1.92 (2H, m) $\delta(^{13}C)$ = 24.7 ppm. Sorangibactin A1 consists of five ring systems, starting from an N-terminal with a salicylic acid, which carbonyl group is fused into an oxazole connected to two consecutive N-methylated thiazolidines linked via an amide bond to a cyclic homocysteine thiolactone C-terminus. The salicylic acid moiety is assigned by corresponding COSY correlations between the four aromatic double bond signals at $\delta(^{1}H)$ = 7.82, 7.36, 7.00 and 6.95 ppm in combination with HMBC correlations to the quaternary carbon with a downfield shift at $\delta(^{13}C)$ = 158.5 ppm due to the hydroxyl substituent and a quaternary carbon, which is deshielded with a shift of $\delta(^{13}C)$ = 112.4 ppm. The remaining aromatic signal exhibits a

characteristic downfield shift at $\delta(^1H)$ = 7.89 ppm with distinct HMBC correlations to two quaternary carbons at $\delta(^{13}C)$ = 162.9 and 144.5 ppm pointing towards a heterocyclic ring with two double bonds to maintain aromatization. The ^1H-^{15}N-HMBC spectrum uncovers the corresponding correlation to a deshielded nitrogen atom with a downfield shift at $\delta(^{15}N)$ = 239.9 ppm characteristic for oxygen containing heteroaromatic systems as oxazole moieties. The linkage between salicylic acid and oxazole is the deshielded quaternary carbon at $\delta(^{13}C)$ = 162.9 ppm due to HMBC correlations from the salicylic acid signal at $\delta(^1H)$ = 7.82 ppm. This carbon atom represents the fused carbonyl group of the salicylic acid. The connection to the adjacent ring is represented by the corresponding COSY and HMBC correlations from the methine signal at $\delta(^1H)$ = 5.01 ppm to the oxazole moiety. Corresponding 2D NMR correlations underpin this ring to be an N-methylated thiazolidine cycle. The diastereotopic methylene signal could be observed at $\delta(^1H)$ = 3.29 and 3.16 ppm as well as the methyl signal at $\delta(^1H)$ = 2.57 ppm with HMBC correlations to the methine signal at $\delta(^1H)$ = 5.01 ppm and the one at $\delta(^1H)$ = 3.29 ppm, respectively. Further on, the COSY spectrum exhibits cross peaks for the latter methine signal to the methine signal of the subsequent N-methylated thiazolidine ring at $\delta(^1H)$ = 4.21 ppm. The corresponding shifts of this ring are more shielded in comparison to the previous are explained by its greater distance to the deshielding oxazole moiety. This is supported by the ^1H-^{15}N HMBC spectrum with corresponding cross peak signals for the nitrogen atom in the third ring at $\delta(^{15}N)$ = 59.8 ppm and the one in the fourth cycle at $\delta(^{15}N)$ = 56.7 ppm. Finally, the fourth and fifth ring system are linked via an amide group with a shift of $\delta(^{13}C)$ = 167.9 in line with HMBC correlations from the thiazolidine signals at $\delta(^1H)$ = 3.59 and 3.15, 3.02 ppm on the one side and the methine signal at $\delta(^1H)$ = 4.09 ppm from the last ring on the other side. This moiety features COSY correlations from a diastereotopic signal at $\delta(^1H)$ = 2.10, 1.92 ppm to the mentioned methine group as well as to another methylene group at $\delta(^1H)$ = 3.01 ppm. Moreover, corresponding HMBC correlations from this methine and diastereotopic methylene signals to the deshielded quaternary carbon with a shift of $\delta(^{13}C)$ = 179.5 ppm indicate a cyclic homocysteine thiolactone C-terminus, which is in line with already published homocysteine thiolactones like thiolactomide.(*4*) The respective deviations between our measurements in methanol-d_4 and the reference data of thiolactomide in dimethylsulfoxid-d_6 are <0.5 ppm for ^1H- and <6 ppm for ^{13}C-shifts. However the quaternary carbon of the thioester shows a greater difference with 26.3 ppm, which can be explained by the different surrounding functions of shielding

thiazolidins and deshielding alkyl chains. Further, the missing correlation in the ^{1}H-^{15}N-HMBC spectrum might be due to a possibly unfavorable conformation for the detection of the ^{3}J(^{1}H-^{15}N)-coupling from the diastereotopic methylene group to the nitrogen atom of the amide bond.

The two signal sets for sorangibactin A1 and A2 were identical except for signals corresponding to the C-terminal homocysteine thiolactone due to isomerization upon purification. The methine signal for sorangibactin A1 can be found at $\delta(^{1}H)$ = 4.09 ppm and for A2 at $\delta(^{1}H)$ = 4.16 ppm, whereby the adjoining diastereotopic methylene group of sorangibactin A1 is located at $\delta(^{1}H)$ = 2.10,1.92 ppm and A2 shows a methylene group signal without any diastereotopic effect at $\delta(^{1}H)$ = 2.04 ppm.

Structure elucidation of sorangibactin B: explained in the main text.

1.8 Marfey's analysis for determination of stereochemistry

To determine the stereochemistry of sorangibactin A, a Marfey's analysis was applied. About 0.1 mg of sorangibactin A1 and A2 mixture was hydrolyzed in 100 µL of 3 N HCl at 110 °C for 45 min. After evaporation of the solvent at 110 °C, two 50 µL aliquots of hydrolyzed sorangibactin A1 and A2 in ddH_2O were supplemented with 60 µL of 1 N $NaHCO_3$ each and 20 µL of the derivatization reagent (1% 1-fluroro-2,4-dinitrophenyl-5-leucine-amide solution in acetone [D-FDLA and L-FDLA]). The obtained mixture was reacted at 40 °C and 700 rpm for 2 h. Addition of 60 µL 3 N HCl stopped the reaction, and the solution was diluted with 150 µL of methanol and acetonitrile to be analyzed by HPLC-HRMS with a Dionex Ultimate 3000 SL system coupled to the maXis4G system. The measurement was done in positive ESI mode after separation by a Waters Acquity BEH C18 column (50 × 2.1 mm, 1.7 µm) with following multi-step gradient (A = 0.1% formic acid in ddH_2O and B = 0.1% formic acid in acetonitrile): 5-10% B in 1 min, 10-35% B in 14 min, 35-55% in 7 min, 55-80% in 3 min, hold at 80% for 1 min and re-equilibration at 5% B for 5 min. The flow was split to 75 µL/min before entering the MS. UV data were recorded at 340 nm simultaneously with the MS detection in centroid mode ranging from *m/z* 150-1000.

1.9 Bioactivity evaluation of sorangibactin A

All microbial strains, except for *M. smegmatis* which was inoculated into M7H9 medium (4.7 g/L Middlebrook 7H9 Broth Base, 4 mL glycerol, 100 mL BD BBL™ Middlebrook OADC Enrichment) and fungi which were inoculated into Myc 2.0 medium (10 g/L Bacto peptone, 10

g/L yeast extract, 20 mL glycerol, pH 6.3), were inoculated into MHB II medium (BD 212322) to achieve a final inoculum of 10^5 colony-forming units (CFU)/mL. Sorangibactin A was prepared as a DMSO stock. Serial dilutions in the growth medium were prepared in a sterile 96-well plate and the strain suspensions were added. Growth inhibition was assessed after incubation for 24 h at RT, 30 °C or 37 °C. Minimum inhibitory concentrations (MIC) are defined as the lowest compound concentration where no visible growth is observed.

Cells were seeded into a sterile CellBIND 96-well plate with DMEM (Gibco 41965039) supplemented with 10% FBS (PAN-Biotech P40-37500) to obtain a final concentration of 10^5 cells/mL. For equilibration, the cells were incubated at 37 °C with 5% CO_2 for 2 h. The prepared sorangibactin A stock in DMSO was added to a separate sterile flat-bottom 96-well plate, serially diluted, and transferred from the lowest to highest concentration to the cell plate. After static incubation at 37 °C with 5% CO_2 for 5 days, the cells were stained with thiazolyl blue tetrazolium bromide (MTT-reagent) at a concentration of 500 µg/mL for 2 h at 37 °C with 5% CO_2. The cells converted the reagent to violet crystals. After discarding the media, hydrochloride/isopropanol (1:250) solution was added to dissolve the crystals. OD was measured at 570 nm and 630 nm as a reference. IC_{50} was assessed by analyzing the nonlinear curve regression.

2. Supplementary tables

Table S1 Annotation of the sorangibactin biosynthetic gene cluster. BLAST was performed using the blastp algorithm with default parameters in the NCBI non-redundant protein sequences database.

Name	Length (aa)	Deduced function	Closest homologue	Coverage/identity (%)
SrbA	317	alpha/beta hydrolase	PYQ66785.1	89/44.70
SrbB	472	salicylate synthase	WP_020740538.1	93/61.88
SrbC	2146	non-ribosomal peptide synthetase	WP_020740537.1	98/65.29
SrbD	1814	non-ribosomal peptide synthetase	KYF49860.1	99/68.72
SrbE	371	saccharopine dehydrogenase NADP-binding domain-containing protein	WP_020740533.1	100/61.89
SrbF	482	bifunctional Gfo/Idh/MocA family oxidoreductase/class I SAM-dependent methyltransferase	WP_020740532.1	80/49.21
SrbG	516	class I SAM-dependent methyltransferase	AGP40737.1	99/54.26
SrbH	394	cytochrome P450	KYG09918.1	99/74.10
SrbI	358	thiazolinyl imide reductase	KYF49855.1	97/74.86
SrbJ	237	VOC family protein	WP_044986807.1	95/67.11
SrbK	538	AMP-binding protein	WP_044986805.1	99/73.64
SrbL	190	TonB-dependent siderophore receptor	WP_245591859.1	55/60.58
SrbM	675	TonB-dependent siderophore receptor	WP_020740545.1	100/66.62
SrbN	407	hypothetical protein	KYF49844.1	90/62.77
SrbO	297	ABC transporter ATP-binding protein	WP_020740540.1	95/79.79
SrbP	1130	ABC transporter permease	KYG09926.1	99/68.62
SrbR	93	LysR family transcriptional regulator	WP_089851301.1	83/45.45

Table S2 Bioactivity results of sorangibactin A (**1** and **2**).

**Microbial strains**	**MIC [µg/ml]**
E. coli acrB [JW0451-2]	64
P. aeruginosa PA14 [DSM-19882]	>64
E. coli WT [BW25113]	>64
B. subtilis [DSM-10]	64
S. aureus [Newman]	64
A. baumannii [DSM-30008]	>64
C. freundii [DSM-30039]	>64
M. hiemalis [DSM-2656]	32
P. anomala [DSM-6766]	64
C. albicans [DSM-1665]	>64
C. neoformans [DSM-11959]	64
M. smegmatis [MC2155]	>64
**Cell line**	**IC_{50} [µg/mL]**
HepG2	8.952 ± 5.65

Table S3 Substrate specificities of adenylation domains in *srb* gene cluster. Prediction was performed by NRPSpredictor2 incorporated in antiSMASH.

**Domain**	**Specificity-conferring code**	**Predicted substrate**	**Actual substrate**
SrbC-A1	DLYNLGLIHK	Cys	Ser
SrbC-A2	DLFNLSMIWK	Cys	Cys
SrbD-A	DLYNLSLIWK	Cys	Cys
SrbK-A	ALPAQGVLNK	Sal	Sal

Table S4 Oligo nucleotides used in this study.

Name	Sequence (5' to 3')	Application
cos-chk-F	TCGTGCTGCTGTCATTGGTGTGC	end-sequencing of cosmid
cos-chk-R	ATAGCGCCCCTGTGTGTTCTCGT	
km-vanR-Pvan-F	ACGAGGCACGCGCTCGCTGCCACCAGGGCAGCACAGAAGACAAGTCTCATCATAATCTGT ACCTCCTTAAGTCAGAAGAACTCGTCAAGAAGGC	promoter engineering
km-vanR-Pvan-R	GCTGCGCCTCGAGCCTCCTGCCCCTGCGCGGACACGGCACACAGGATTCCCATATGCGTT TCCTCGCATCG	
km-vanR-Pvan-delA-R	ACGTACGTCGGCCTATCGGACATACCGTCCTCAGCGTACTGTCGATATTTCATATGCGTTTC CTCGCATCG	knockout of *srbA*
km-vanR-Pvan-delAB-R	AGCACGCGGGCGATCGTGCCCCGCACATCCAGGAAACTATCGTGACTCGGCATATGCGTT TCCTCGCATCG	knockout of both *srbA* and *srbB*
amp-Ptet-delE-F	TCATCGCCGTGCTCGGTGCTTACGGCGATGTGGGGCGCCAGGCCACGCGGTTACCAATGC TTAATCAGTGAGG	knockout of *srbE*
amp-Ptet-delE-R	CGTCTCGACCAGCACGGAGATCCCTCGTTCGAGCTGCTCGCCGATGCTCATAGATCCTTTC TCCTCTTTAGATC	
amp-Ptet-delF-F	GCATCGGCGAGCAGCTCGAACGAGGGATCTCCGTGCTGGTCGAGACGCCGTTACCAATGC TTAATCAGTGAGG	knockout of *srbF*
amp-Ptet-delF-R	TCGAGGGTCCGGCACCACGCGGCGAGCAAGGCCGAGAAGTCGATGATCATAGATCCTTTC TCCTCTTTAGATC	
amp-Ptet-delG-F	TCATCGACTTCTCGGCCTTGCTCGCCGCGTGGTGCCGGACCCTCGATCCGTTACCAATGCT TAATCAGTGAGG	knockout of *srbG*
amp-Ptet-delG-R	TCGATGCCCTCGAAGTCGTTGAACGGATACGGTCGGGGGGAGTCCGTCATAGATCCTTTCT CCTCTTTAGATC	
amp-Ptet-delH-F	CTCGACCAAAAGGATTCGACCCATGACGGACTCCCCCCGACCGTATCCGTTTACCAATGCT TAATCAGTGAGG	knockout of *srbH*
amp-Ptet-delH-R	AAGGTAAATGCGGCCAAAGGCCGTGCCCGCGACGAGGACGCGACGTTTCATAGATCCTTT CTCCTCTTTAGATC	
amp-Ptet-delI-F	AACGTCGCGTCCTCGTCGCGGGCACGGCCTTTGGCCGCATTTACCTTGCCTTACCAATGCT TAATCAGTGAGG	knockout of *srbI*
amp-Ptet-delI-R	TGGTGAATGTCGCGAACCCAGAGCAAAACGTGGCTACAGGACGTGGTCATAGATCCTTTCT CCTCTTTAGATC	
amp-Ptet-delJ-F	CCACGTTTTGCTCTGGGTTCGCGACATTCACCAGGCGGTGCGCGACTACCTTACCAATGCT TAATCAGTGAGG	knockout of *srbJ*
amp-Ptet-delJ-R	CGGTACGTCGCCGCGAACTCCGCAGGCCAATTTACGAAGCCTTCGAGCATAGATCCTTTCT CCTCTTTAGATC	
amp-Ptet-delK-F	ATTGGCCTGCGGAGTTCGCGGCGACGTACCGCGAACTCGGCTACTGGCGTTTACCAATGC TTAATCAGTGAGG	knockout of *srbK*
amp-Ptet-delK-R	CCGCGAGGCTGCGCGGCGATCCGTGCGGCCAGCTCGCGCTTGTTGACCTTAGATCCTTTC TCCTCTTTAGATC	
TE-C1586A-F	CGCAGCGCCTCGTGGACGAGGGCTTCGAAGAGTTCCAGCTCATTGGCTATGCGCTCGGCG GGCTGCTGGCCATGTTTAAACTTTGTTCAAAAAAAA	TE point mutation
TE-C1586S-F	CGCAGCGCCTCGTGGACGAGGGCTTCGAAGAGTTCCAGCTCATTGGCTATTCGCTCGGCG GGCTGCTGGCCATGTTTAAACTTTGTTCAAAAAAAA	
TE-C1586-R	CCGCGCTCGAGCAGACGGCGCGCCACCTCGATGGCCAGCAGCCCGCCGAGGTTTAAACT TTGTTTATTTTTCT	
srb-chk01-F	CCAAGTACAAGCAGCTCGTCGCA	colony PCR
srb-chk01-R	GCCGTCGTCGTCGATGAACATCA	
srb-chk02-F	CTACCGCATCCAAGTCGAAGACC	
srb-chk02-R	CGATGCAGGATCGTGAGGTTCAC	
srb-chk03-F	TGGCTTCGATTACCGCACGTTGG	
srb-chk03-R	GGCGTCCTTGGGTATCGAATACG	

Table S5 NMR spectroscopic data of sorangibactin A1 (**1**) in methanol-d_4 at 700/175 MHz.

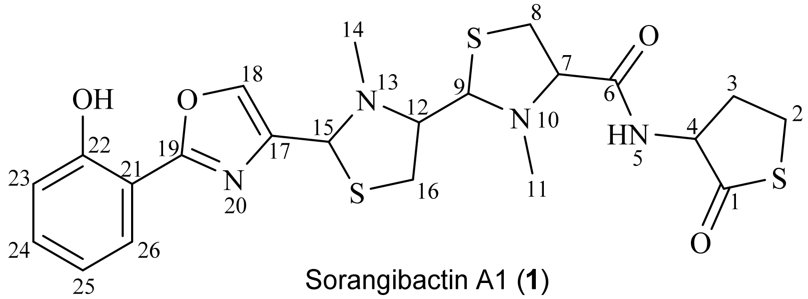

Sorangibactin A1 (**1**)

#	δ ^{13}C [ppm]	δ ^{1}H [ppm] mult (J [Hz])	δ ^{15}N [ppm]	COSY	HMBC	^{1}H-^{15}N HMBC
**1**	179.5	-	-	-	-	-
**2**	24.9	3.01, m	-	3	3,4	-
**3**	24.7	2.10,1.92, m	-	2,4	1,2,4	-
**4**	63.1	4.09, dd (6.94,3.66)	-	3	1,2,6	-
**5**	-	-	-	-	-	-
**6**	167.9	-	-	-	-	-
**7**	81.2	3.59, t (7.95)	-	8	6,8,9,11	-
**8**	35.3	3.15,3.02, m	-	7	6,7,9	10
**9**	76.0	4.21, m	-	12,16	11,16	-
**10**	-	-	56.7	-	-	-
**11**	40.8	2.47, s	-	-	7,9	10
**12**	75.7	3.29, m	-	9,16	9,16	-
**13**	-	-	59.8	-	-	-
**14**	41.9	2.57, s	-	-	12,15	13
**15**	70.1	5.01, s	-	18	12,14,16,17,18	20
**16**	33.8	3.29,3.16, m	-	9,12	14,15	13
**17**	144.5	-	-	-	-	-
**18**	136.4	7.89, s	-	15	17,19	20
**19**	162.9	-	-	-	-	-
**20**	-	-	239.9	-	-	-
**21**	112.4	-	-	-	-	-
**22**	158.5	-	-	-	-	-
**23**	118.2	7.00, d (8.38)	-	24	21,22,25	19,20
**24**	133.7	7.36, m	-	23,25	22,26	19,20
**25**	120.7	6.95, m	-	24,26	21,22,23,24,26	-
**26**	127.2	7.82, d (7.80)	-	25	19,22,24	-

Table S6 NMR spectroscopic data of sorangibactin A2 (**2**) in methanol-d_4 at 700/175 MHz.

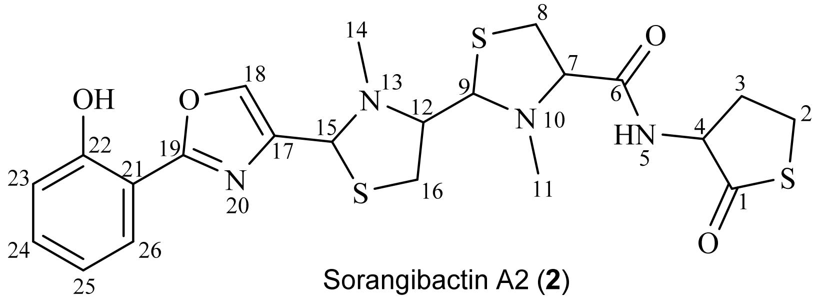

Sorangibactin A2 (**2**)

#	δ ^{13}C [ppm]	δ ^{1}H [ppm] mult (*J* [Hz])	δ ^{15}N [ppm]	COSY	HMBC	^{1}H-^{15}N HMBC
**1**	179.5	-	-	-	-	-
**2**	24.9	3.01, m	-	3	4	-
**3**	23.9	2.04, m	-	2,4	1,2,4	-
**4**	63.1	4.16, dd (6.79,4.29)	-	3	1,2,6	-
**5**	-	-	-	-	-	-
**6**	167.9	-	-	-	-	-
**7**	81.2	3.59, t (7.95)	-	8	6,8,9,11	-
**8**	35.3	3.15,3.02, m	-	7	6,7,9	10
**9**	76.0	4.21, m	-	12,16	11,16	-
**10**	-	-	56.7	-	-	-
**11**	40.8	2.47, s	-	-	7,9	10
**12**	75.7	3.29, m	-	9,16	9,16	-
**13**	-	-	59.8	-	-	-
**14**	41.9	2.57, s	-	-	12,15	13
**15**	70.1	5.01, s	-	18	12,14,16,17,18	20
**16**	33.8	3.29,3.16, m	-	9,12	14,15	13
**17**	144.5	-	-	-	-	-
**18**	136.4	7.89, s	-	15	17,19	20
**19**	162.9	-	-	-	-	-
**20**	-	-	239.9	-	-	-
**21**	112.4	-	-	-	-	-
**22**	158.5	-	-	-	-	-
**23**	118.2	7.00, d (8.38)	-	24	21,22,25	19,20
**24**	133.7	7.36, m	-	23,25	22,26	19,20
**25**	120.7	6.95, m	-	24,26	21,22,23,24,26	-
**26**	127.2	7.82, d (7.80)	-	25	19,22,24	-

Table S7 NMR spectroscopic data of sorangibactin B (**3**) in methanol-d_4 at 500/125 MHz.

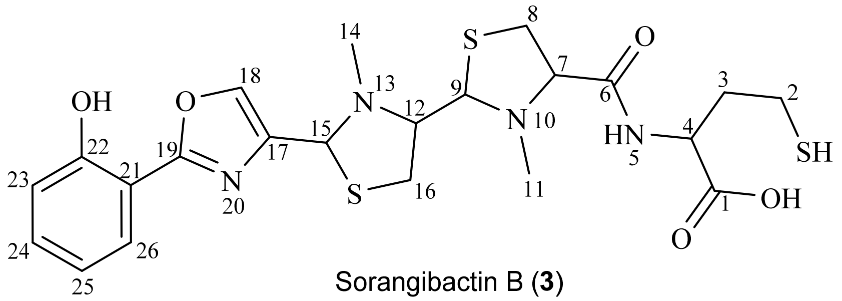

Sorangibactin B (**3**)

#	δ ^{13}C [ppm]	δ ^{1}H [ppm] mult (*J* [Hz])	COSY	HMBC
**1**	176.6	-	-	-
**2**	35.2	2.27, t (7.47)	3	3,4
**3**	26.3	1.59, m	2,4	1
**4**	51.0	4.68, m	3	6
**5**	-	-	-	-
**6**	173.0	-	-	-
**7**	76.3	3.57, m	8	6
**8**	33.7	3.25,3.07, m	7	6,7
**9**	78.1	4.31, m	12	12,14
**10**	-	-	-	-
**11**	42.5	2.56, m	-	7,9
**12**	75.2	3.37, m	9,16	-
**13**	-	-	-	-
**14**	42.5	2.64, m	-	12,15
**15**	69.9	5.06, m	18	17,18
**16**	34.6	3.23,3.08, m	12	12
**17**	142.5	-	-	-
**18**	136.4	7.88, m	15	17,19
**19**	161.8	-	-	-
**20**	-	-	-	-
**21**	110.9	-	-	-
**22**	157.2	-	-	-
**23**	118.2	7.01, m	24	21,22
**24**	133.9	7.38, m	23,25	21,22
**25**	120.8	6.97, m	24,26	21,23
**26**	127.2	7.83, m	25	19,22,24

3. Supplementary figures

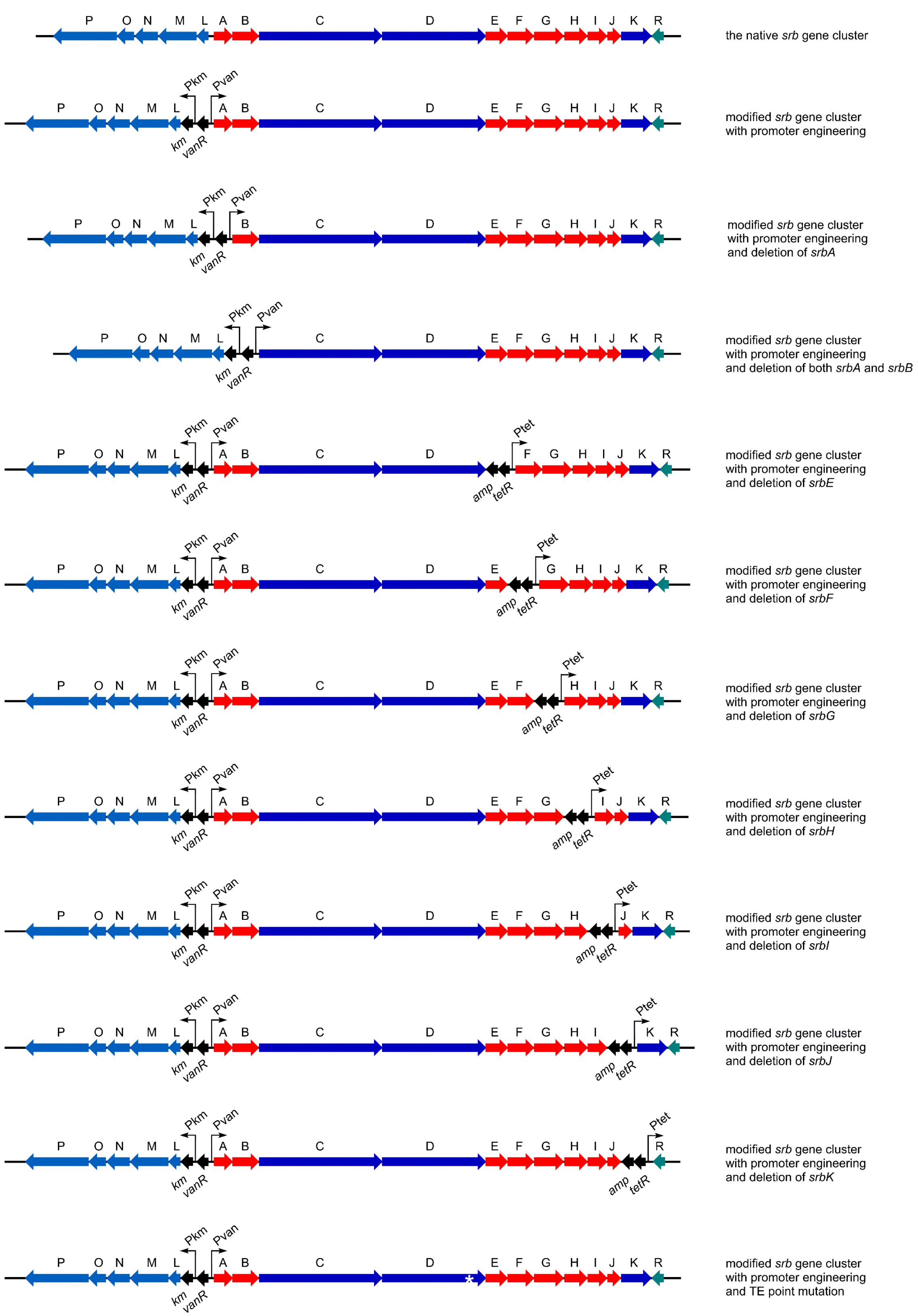

Figure S1 Expression constructs used in this study.

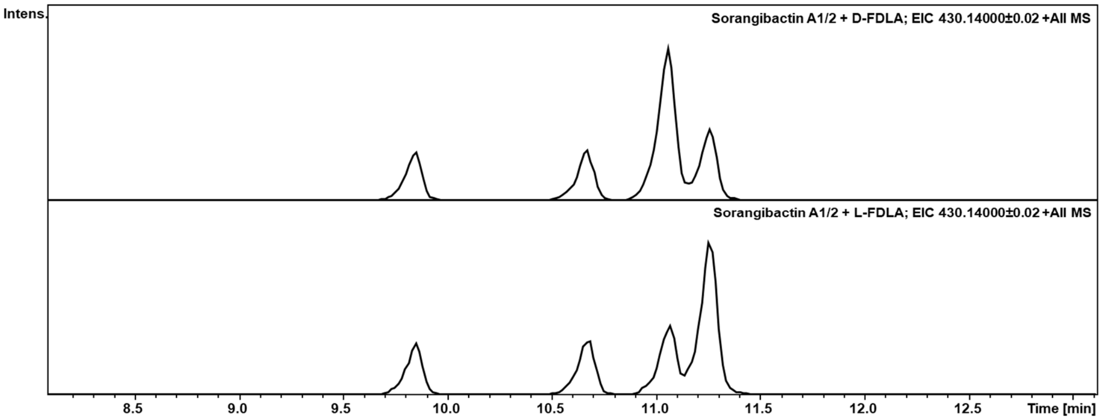

Figure S2 HPLC-MS measurements of Marfey's derivatization of sorangibactin A (**1** and **2**). Upper chromatogram: derivatized with D-FDLA; Lower chromatogram: derived with L-FDLA; The traces are shown as EIC 430.14 ± 0.02 *m/z* for derivatized N-methyl cysteine and homocysteine.

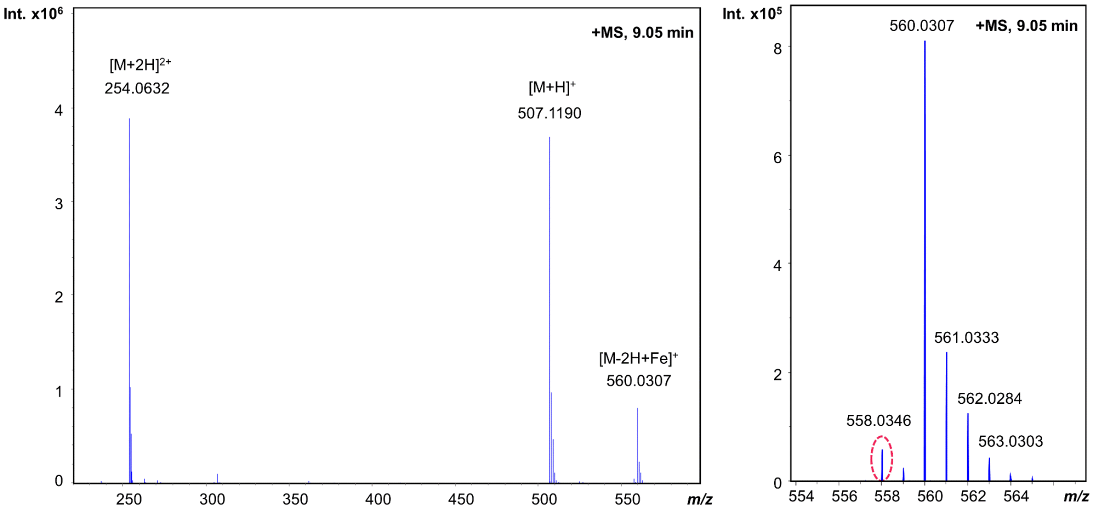

Figure S3 HRMS spectrum of sorangibactin A. UHR-TOF measurements of sorangibactin A containing extract showing the siderophore-like behavior by insource iron chelating: double charged species at *m/z* 254.0632, single charged species at *m/z* 507.1189 and $[M-2H+Fe^{3+}]^+$ species at *m/z* 560.0307 with a characteristic M-2 iron isotope peak at *m/z* 558.0346.

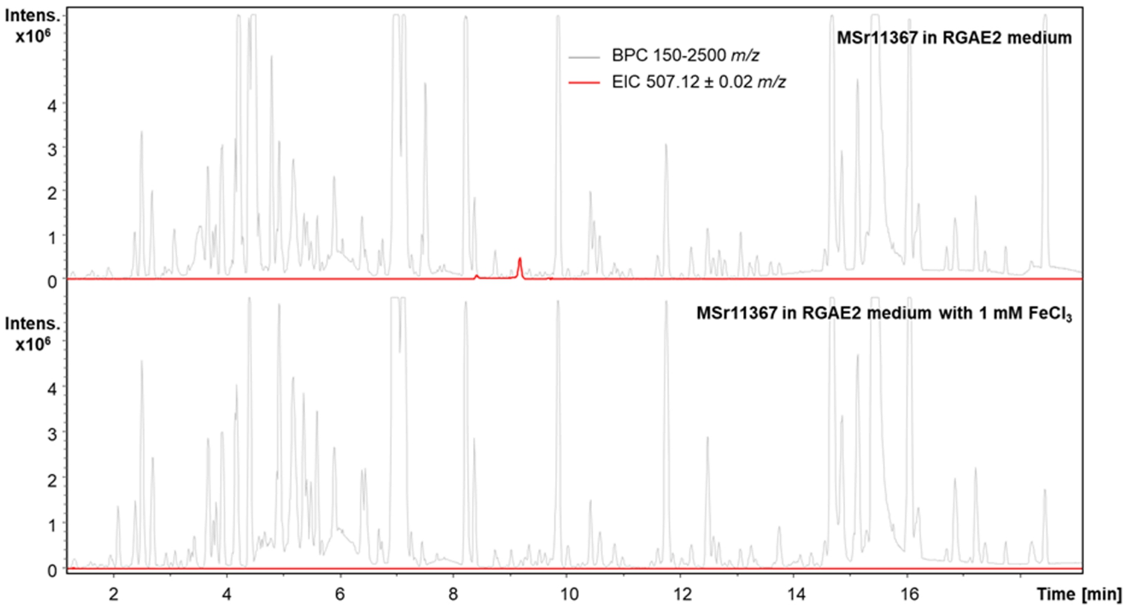

Figure S4 Production of sorangibactin A by MSr11367 with and without iron addition.

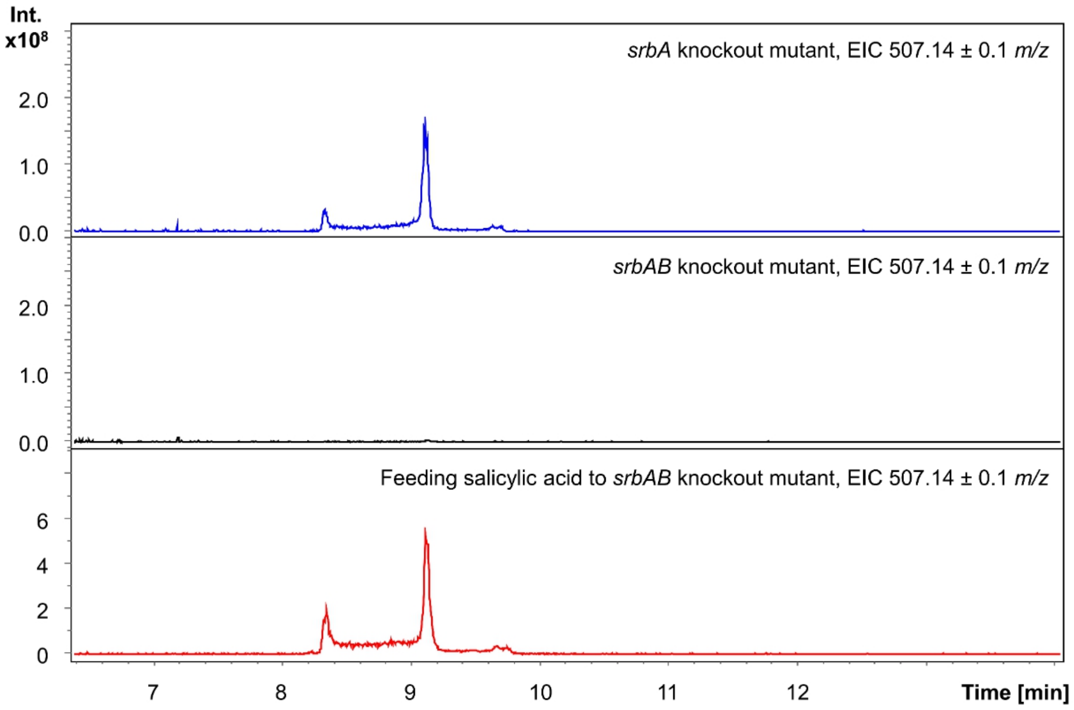

Figure S5 HPLC-MS measurements of *srbA*, *srbAB* knockout mutants and feeding salicylic acid to *srbAB* knockout mutant.

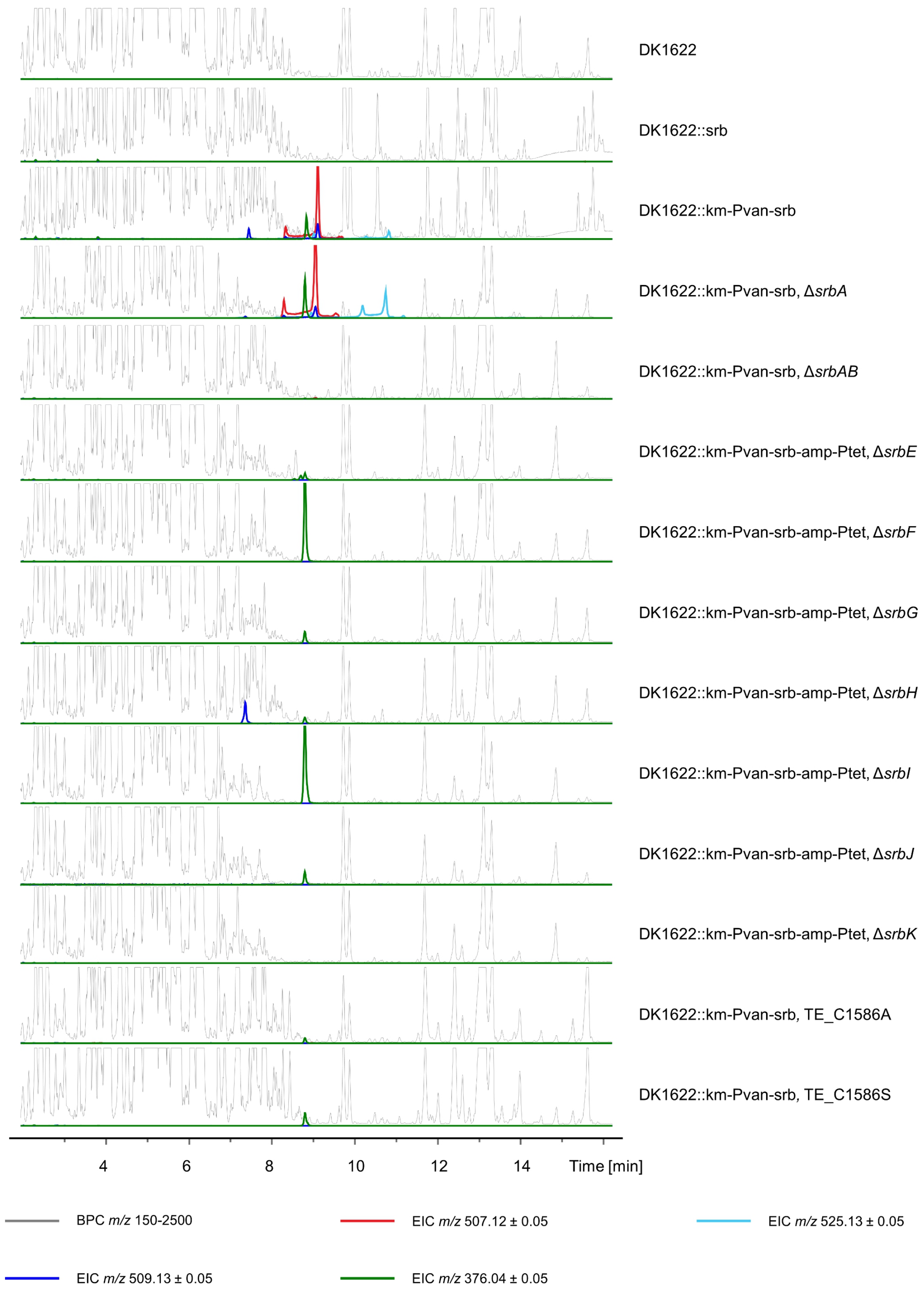

Figure S6 HPLC-MS analysis of crude extrudes from *M. xanthus* DK1622 and mutants containing the corresponding sorangibactin expression constructs.

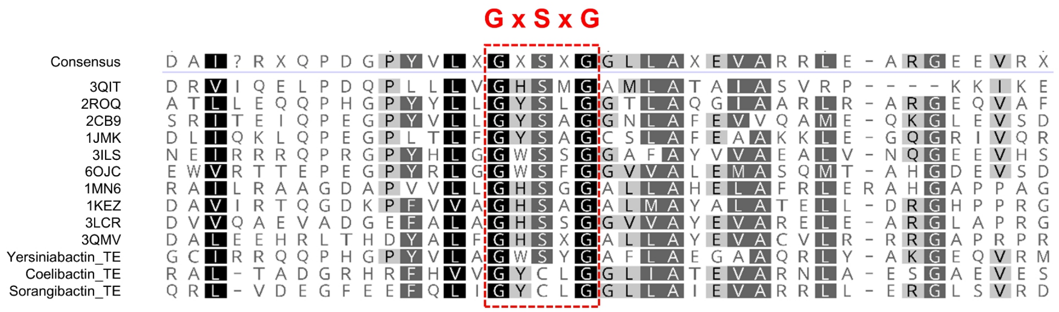

Figure S7 Alignment of TE domains. The sorangibactin TE domain is aligned with coelibactin TE, yersiniabactin TE and 10 structure available TEs from PDB database. The catalytic motif (normally GxSxG) is indicated in red dashed box.

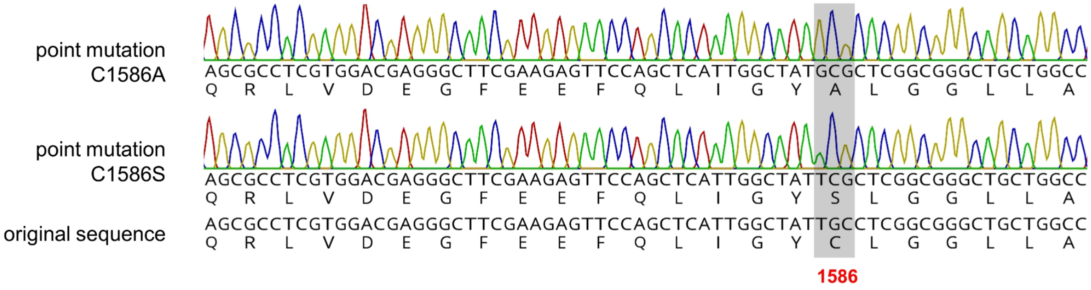

Figure S8 Point mutation of C1586A and C1586S of the sorangibactin TE domain.

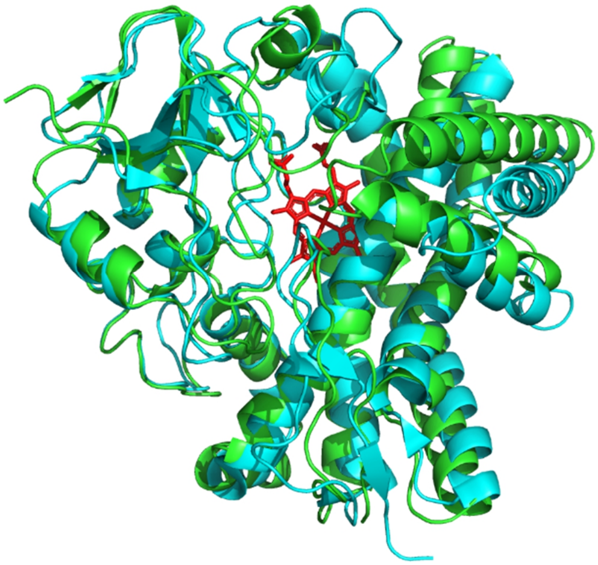

Figure S9 Superimposition of SrbH and CYP105N1. Green color: structure of SrbH predicted by ColabFold;(*5*) Cyan color: CYP105N1 from *Streptomyces coelicolor* A3(2) (PDB 3TYW); Heme is shown as red stick.

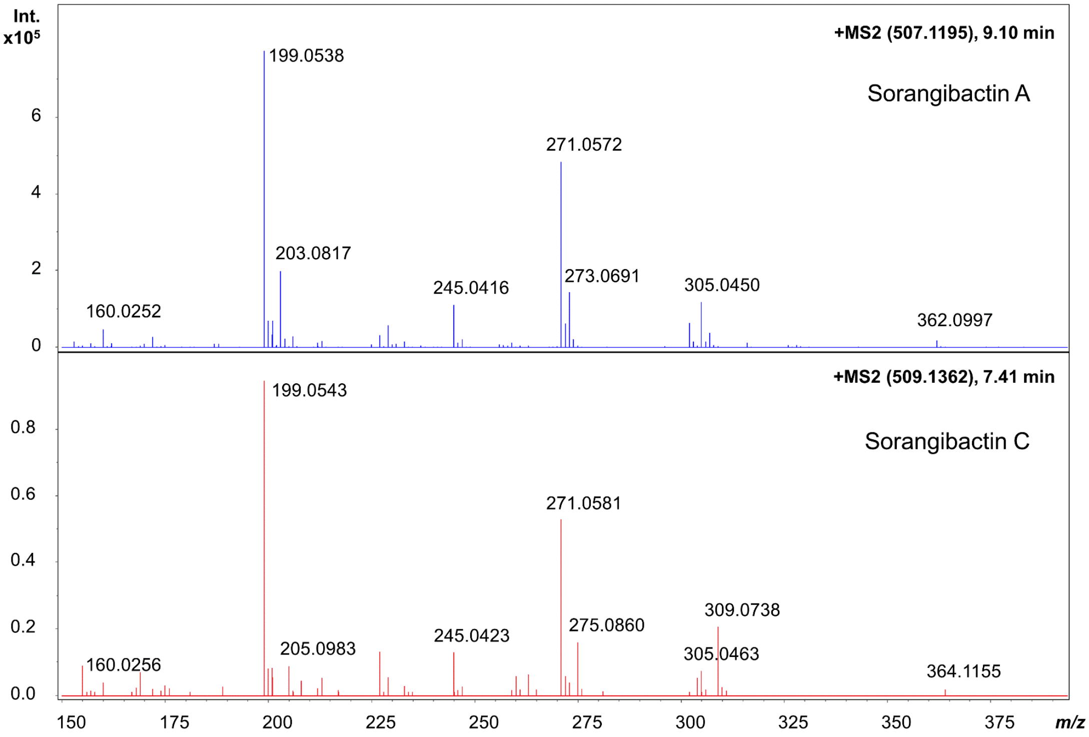

Figure S10 HRMS^2 of sorangibactin C in comparison with sorangibactin A.

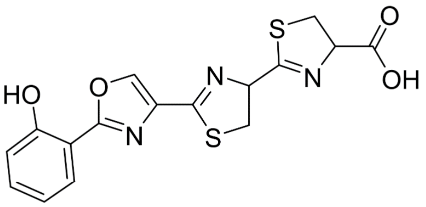

Proposed structure of sorangibactin D
Chemical Formula: $C_{16}H_{13}N_3O_4S_2$
Exact Mass: 375.0347

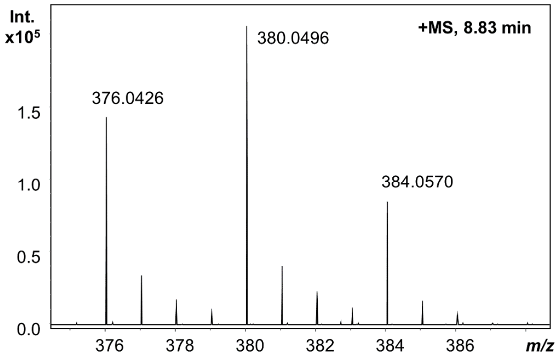

Figure S11 Proposed structure and incorporation of L-Cys-$^{13}C_3$,^{15}N of sorangibactin D.

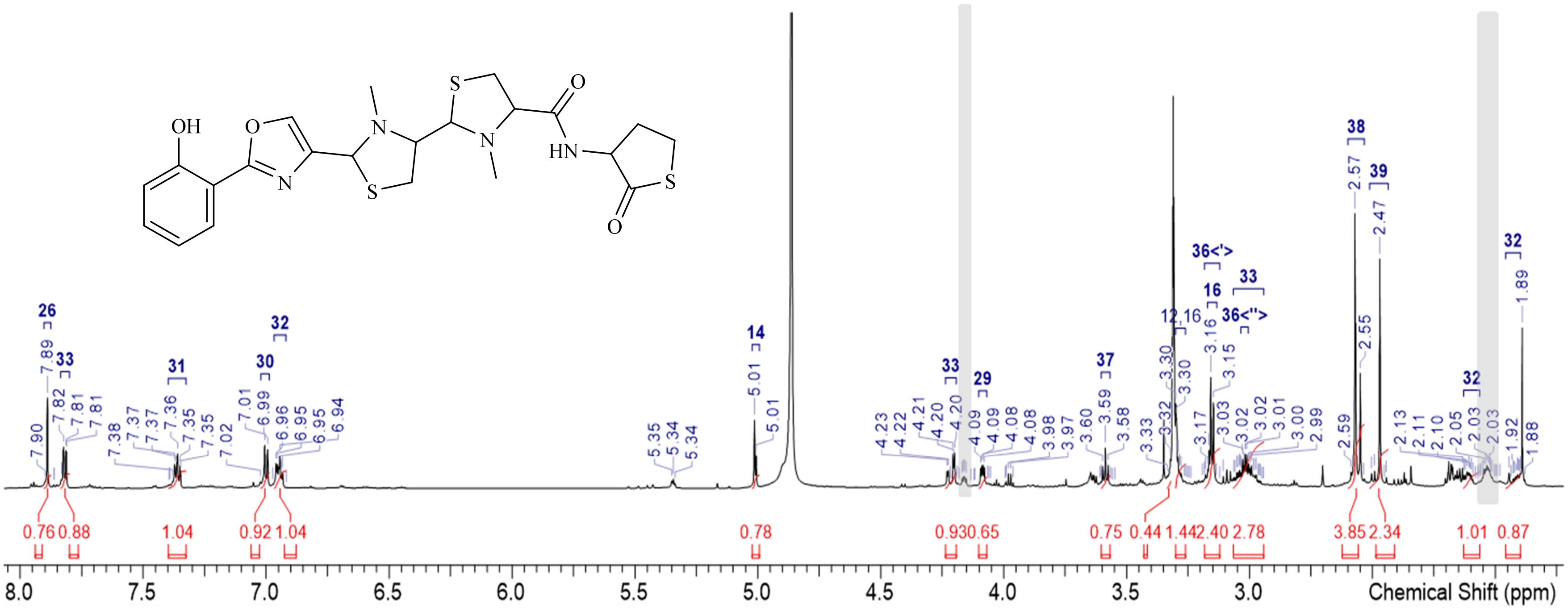

Figure S12 ¹H-spectrum of sorangibactin A1 (**1**) in methanol-d_4 at 700 MHz. Signals corresponding to sorangibactin A2 (**2**) are highlighted in gray.

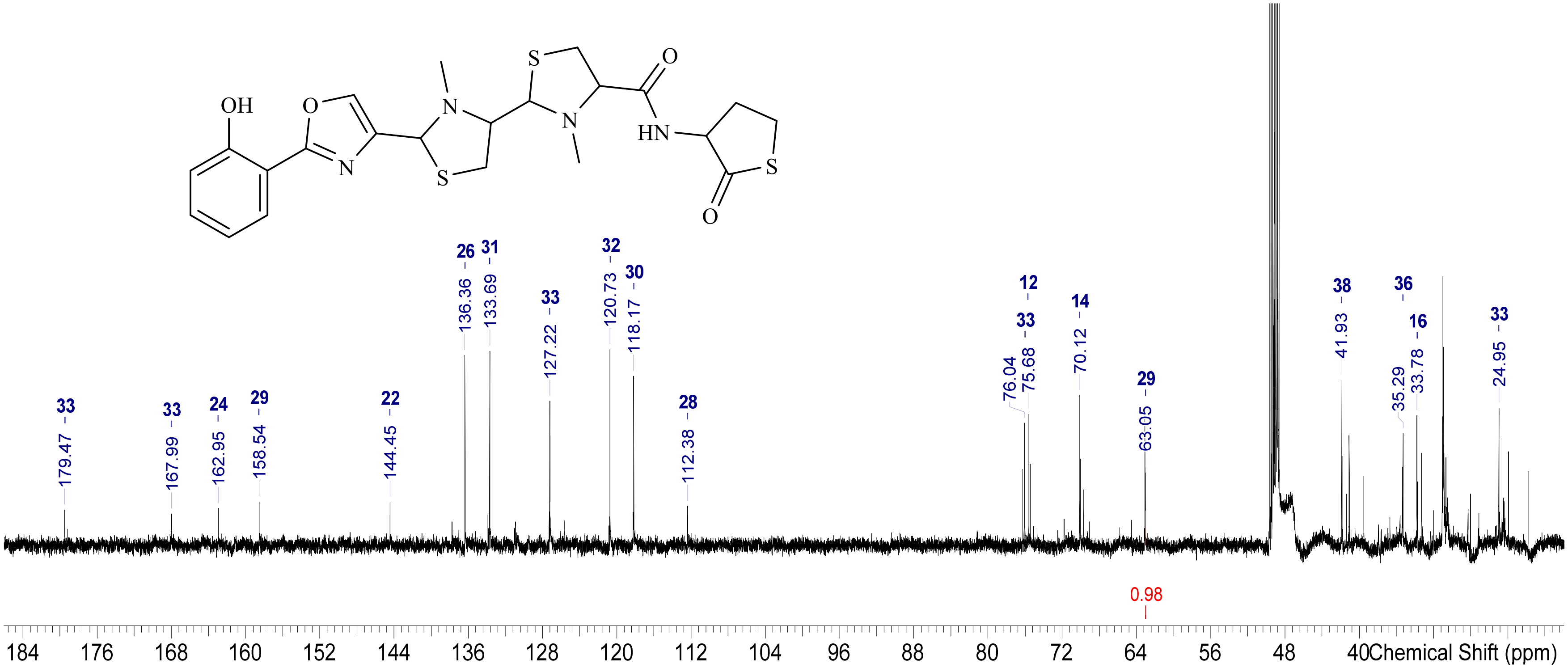

Figure S13 ^{13}C-spectrum of sorangibactin A1 (**1**) in methanol-d_4 at 175 MHz.

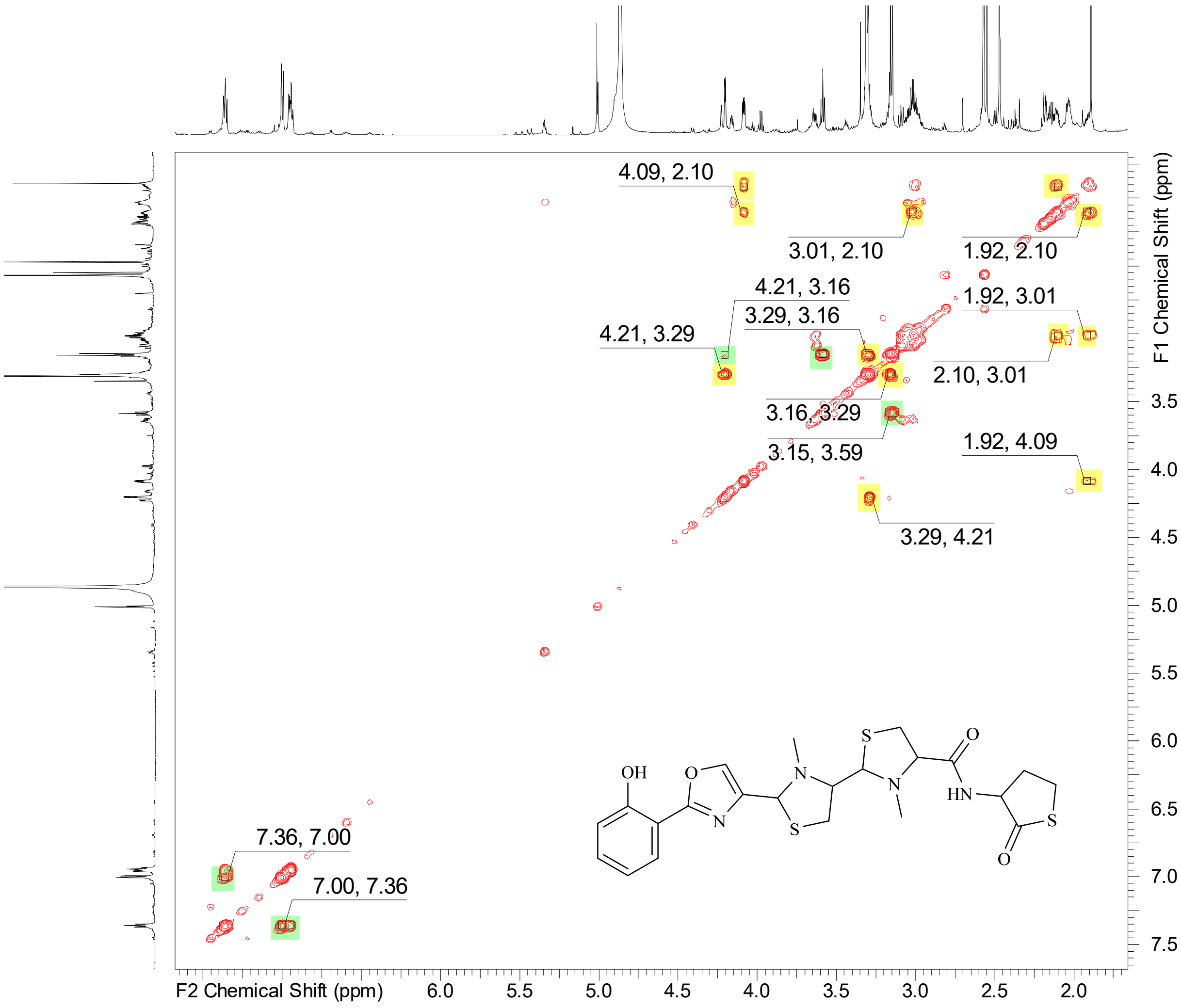

Figure S14 COSY spectrum of sorangibactin A1 (**1**) in methanol-d_4 at 700 MHz.

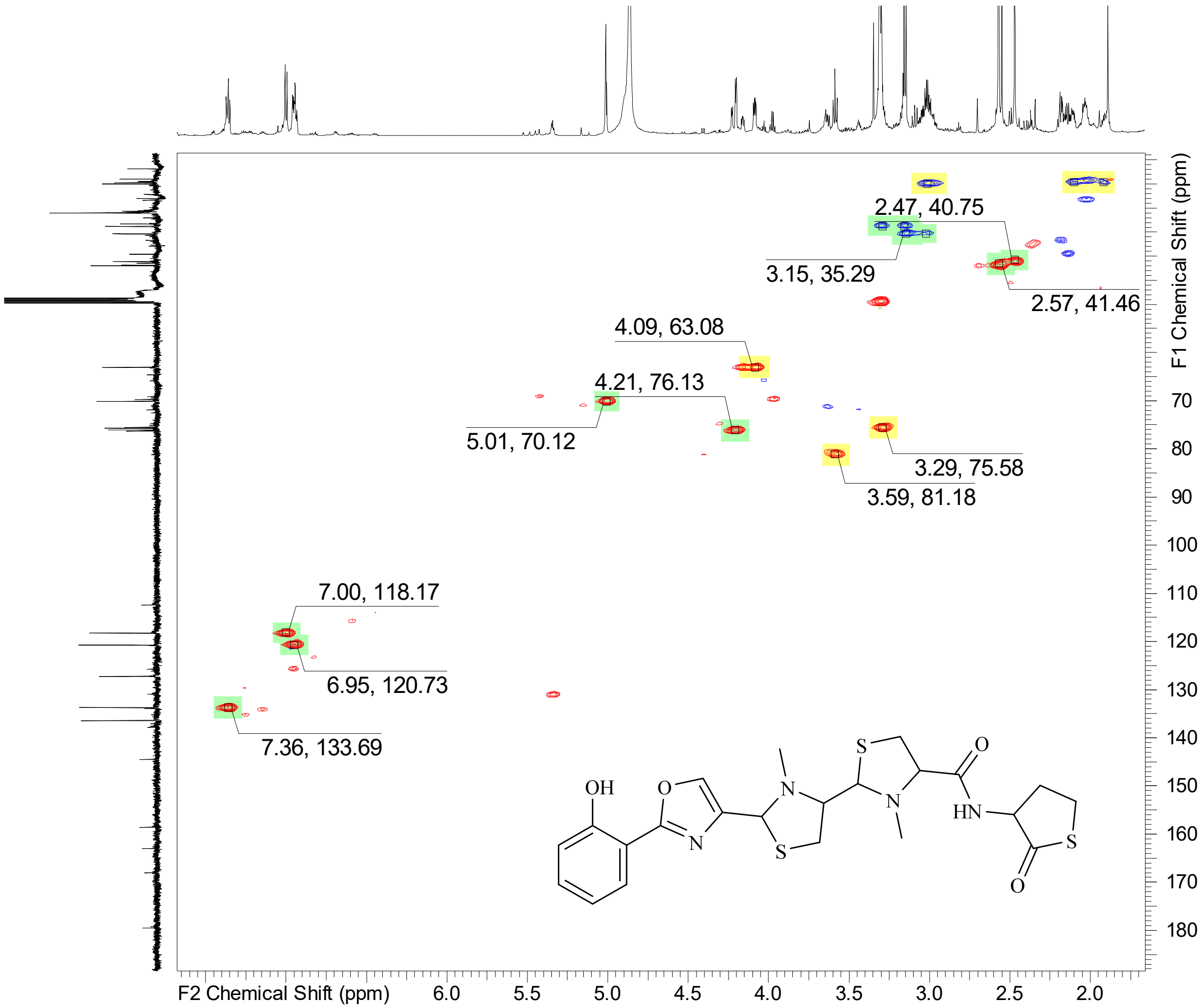

Figure S15 HSQC spectrum of sorangibactin A1 (**1**) in methanol-*d*$_4$ at 700 MHz (^{1}H)/175 MHz (^{13}C).

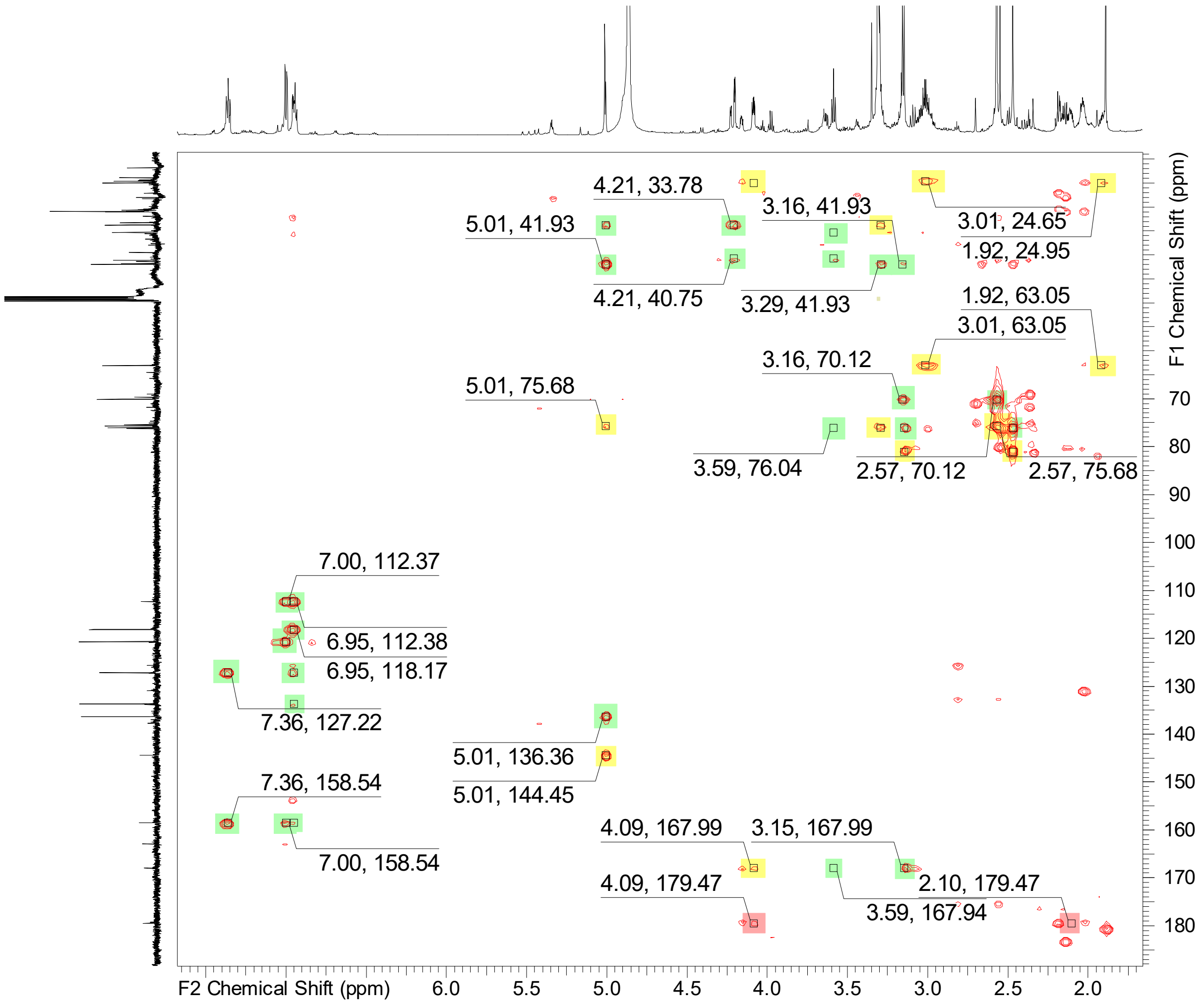

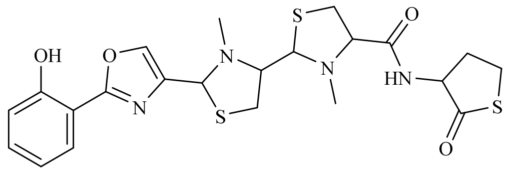

Figure S16 HMBC spectrum of sorangibactin A1 (**1**) in methanol-d_4 at 700 MHz (^{1}H)/175 MHz (^{13}C).

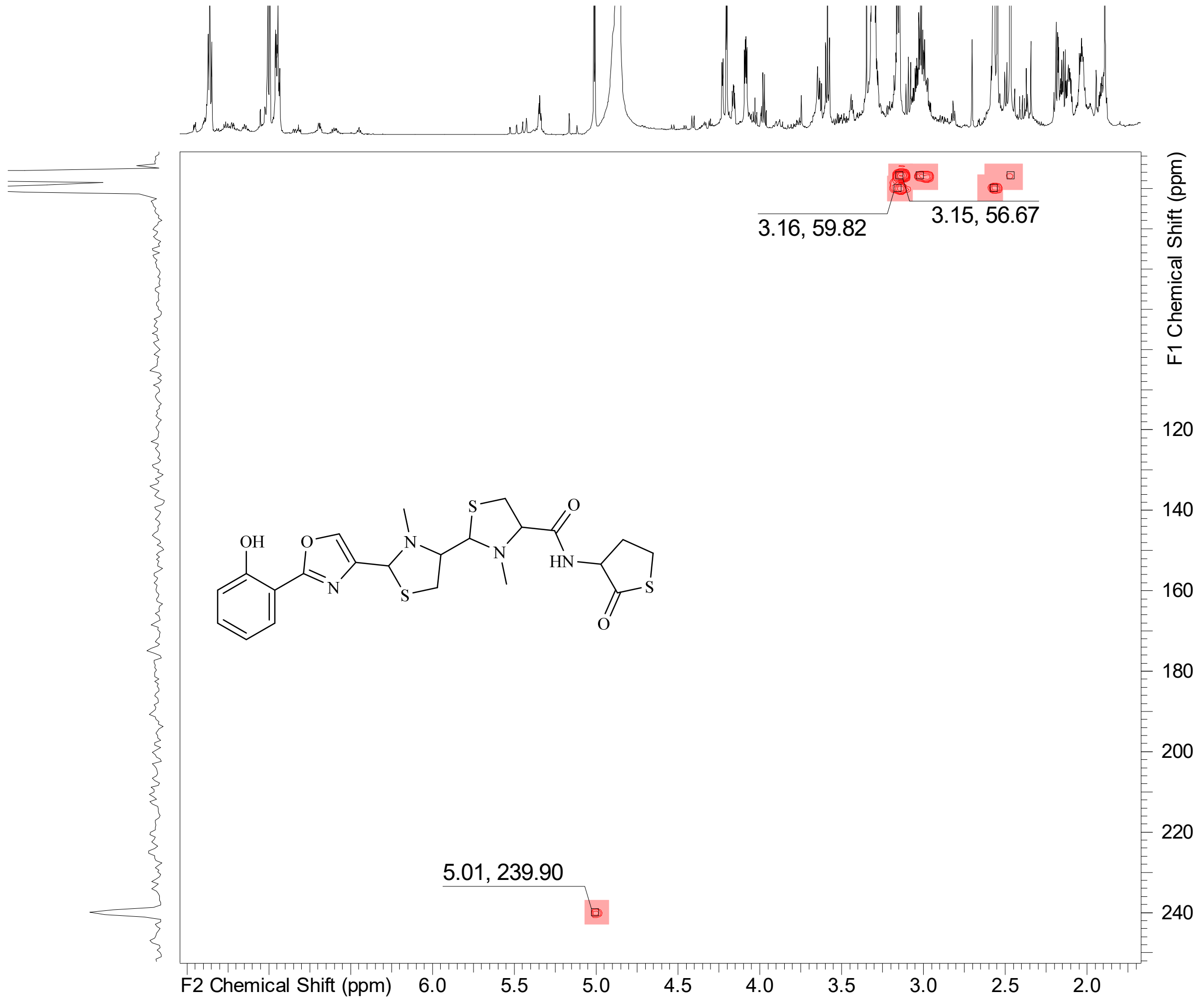

Figure S17 ^{1}H-^{15}N-HMBC spectrum of sorangibactin A1 (**1**) in methanol-d_4 at 500 MHz (^{1}H)/125 MHz (^{15}N).

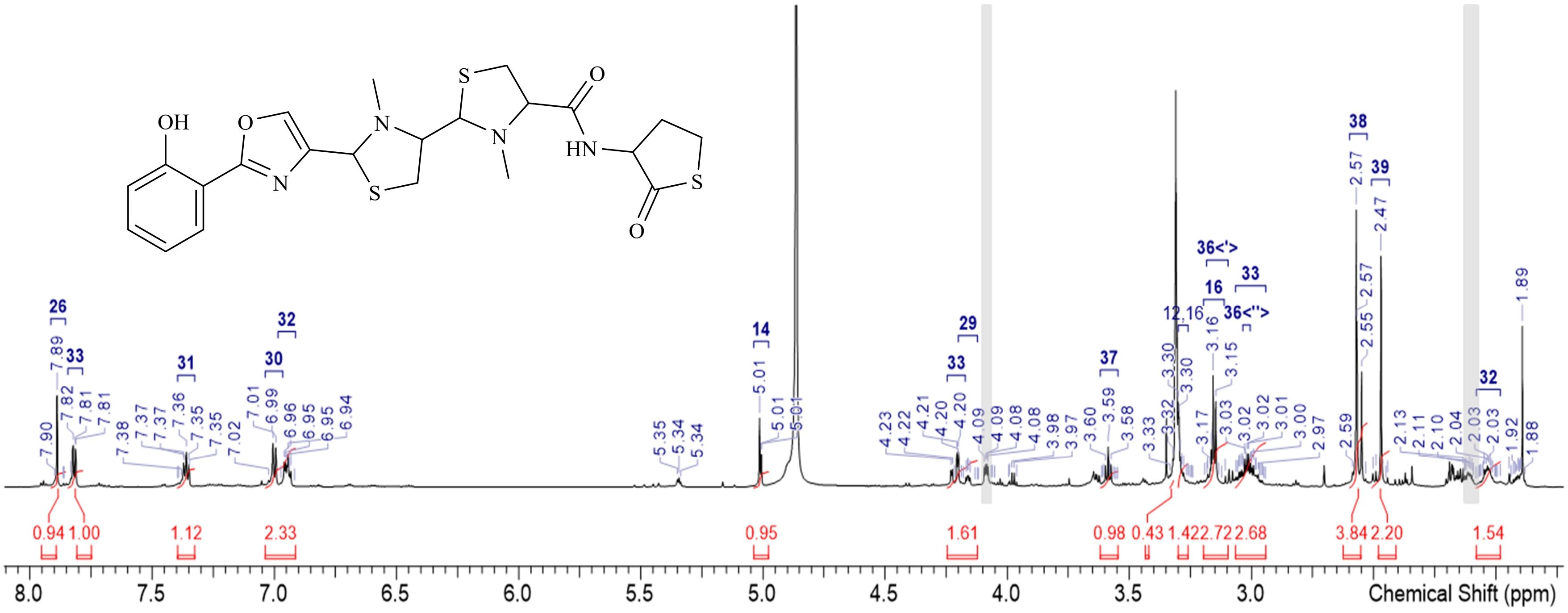

Figure S18 ^{1}H-spectrum of sorangibactin A2 (**2**) in methanol-d_4 at 700 MHz. Signals corresponding to sorangibactin A1 (**1**) are highlighted in gray.

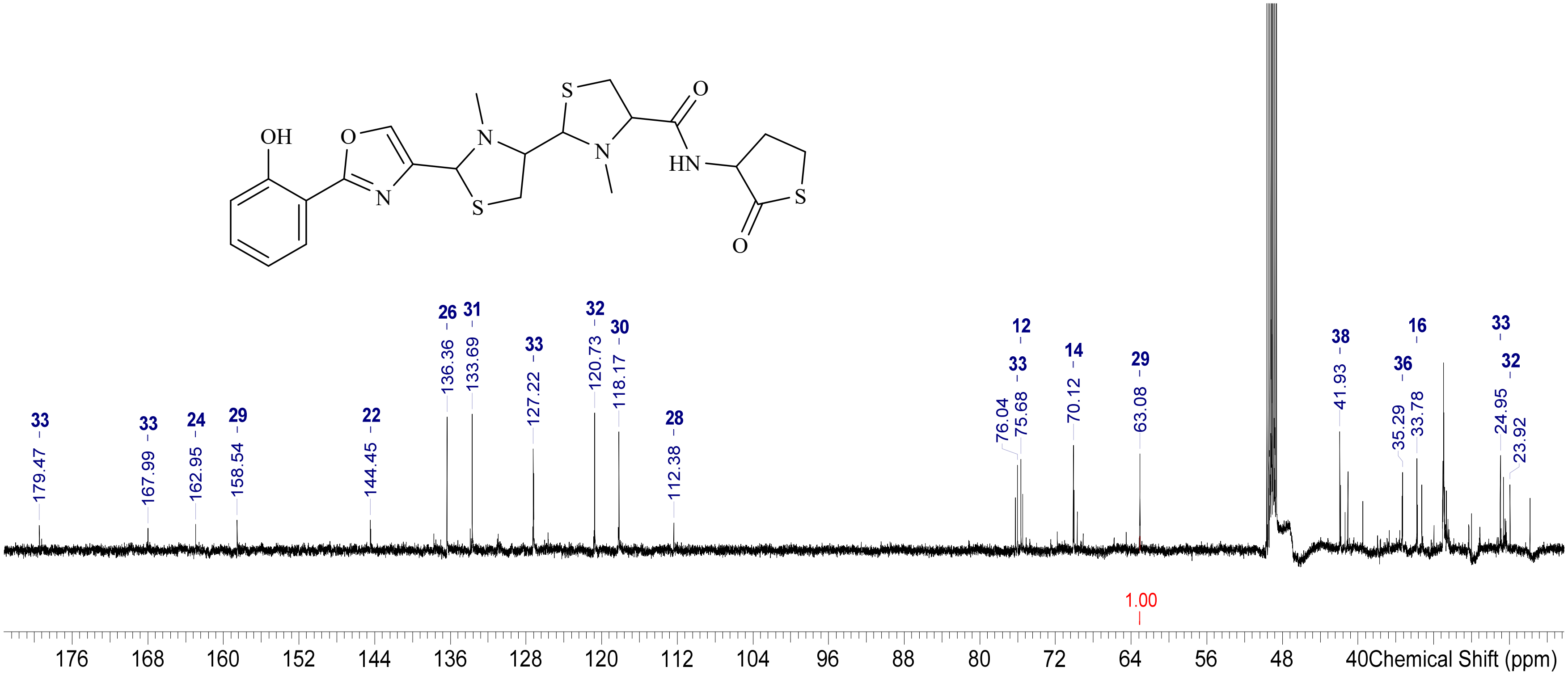

Figure S19 ^{13}C-spectrum of sorangibactin A2 (**2**) in methanol-d_4 at 175 MHz.

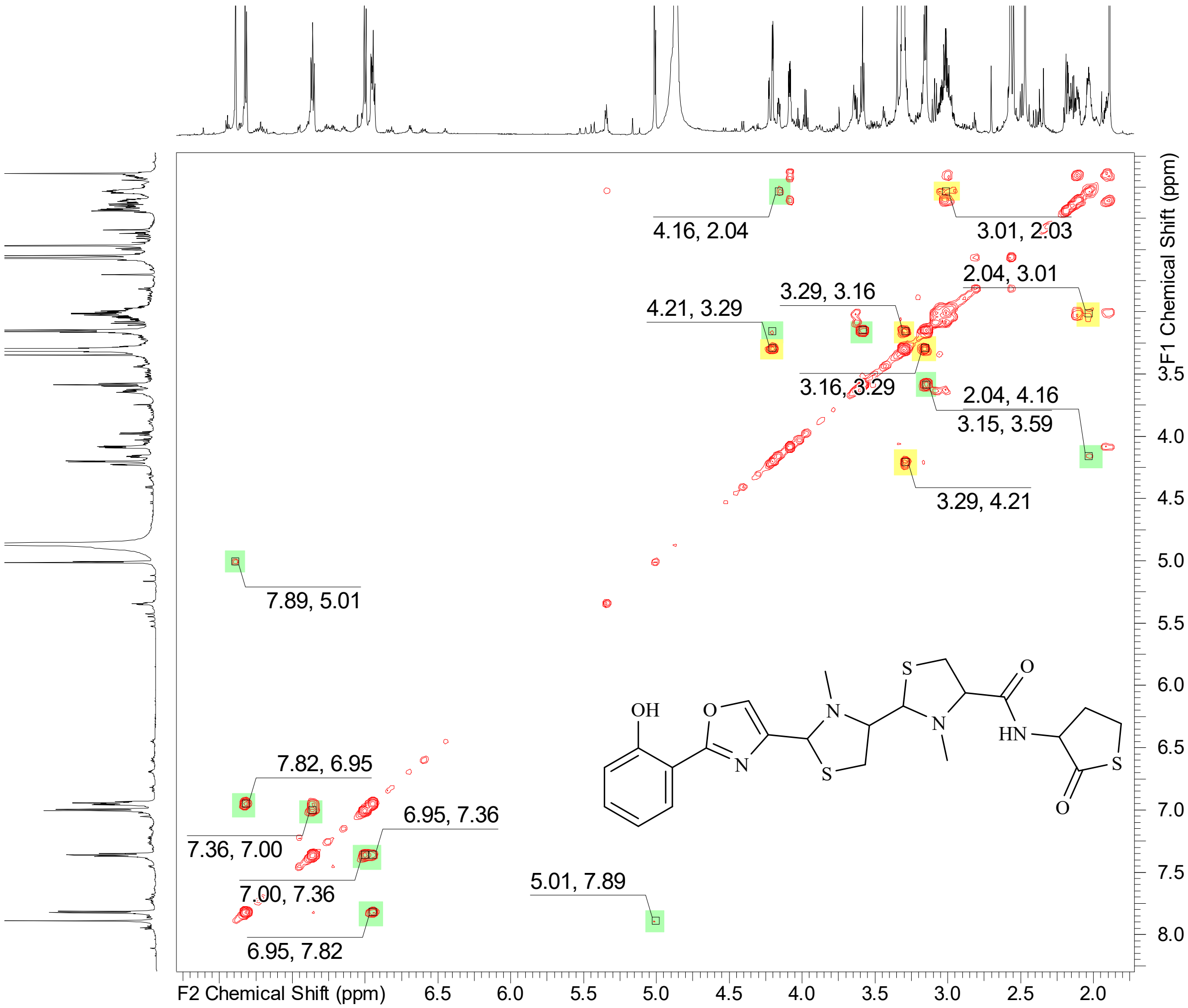

Figure S20 COSY spectrum of sorangibactin A2 (**2**) in methanol-d_4 at 700 MHz.

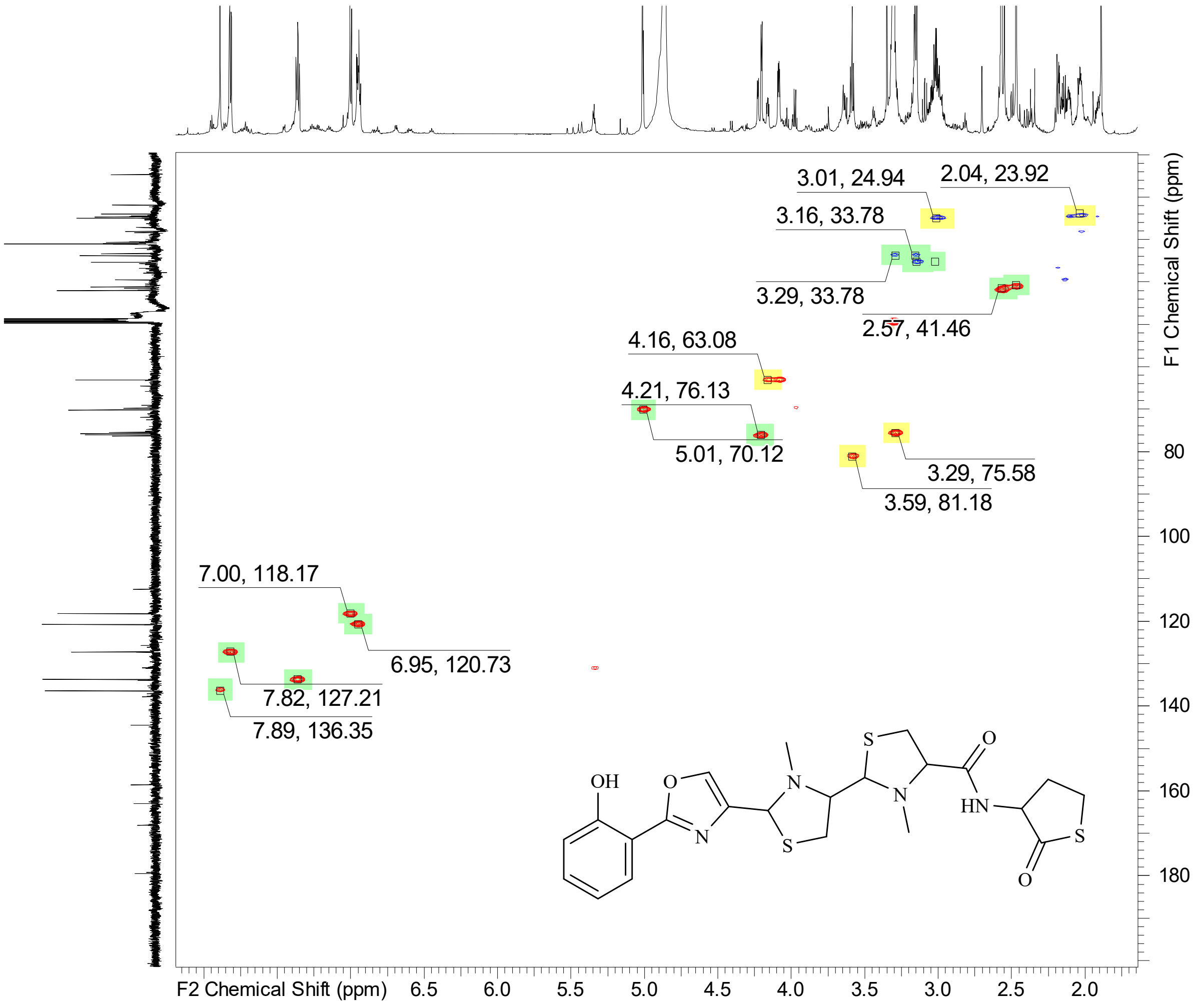

Figure S21 HSQC spectrum of sorangibactin A2 (**2**) in methanol-d_4 at 700 MHz (^{1}H)/175 MHz (^{13}C).

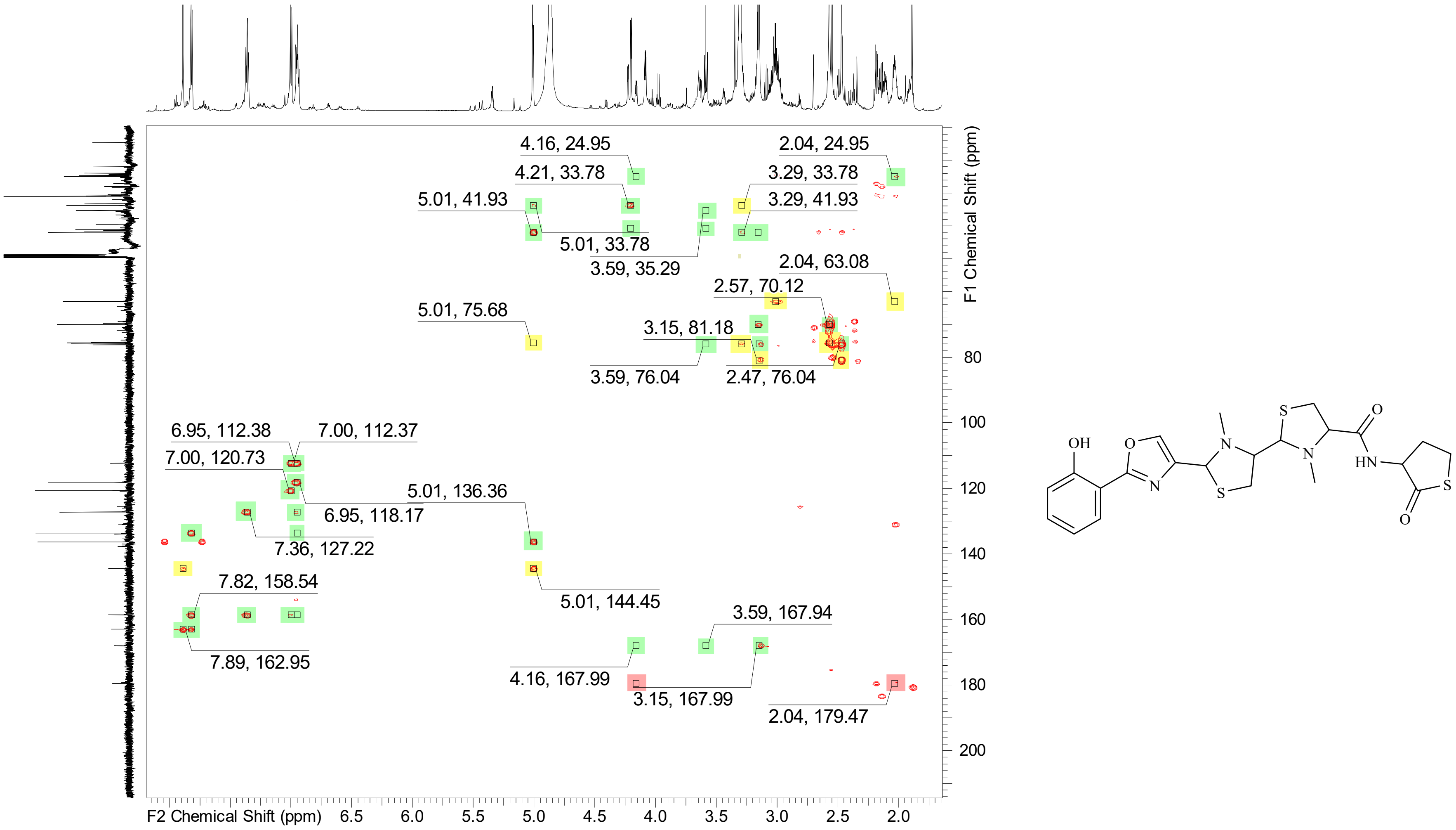

Figure S22 HMBC spectrum of sorangibactin A2 (**2**) in methanol-d_4 at 700 MHz (^1H)/175 MHz (^{13}C).

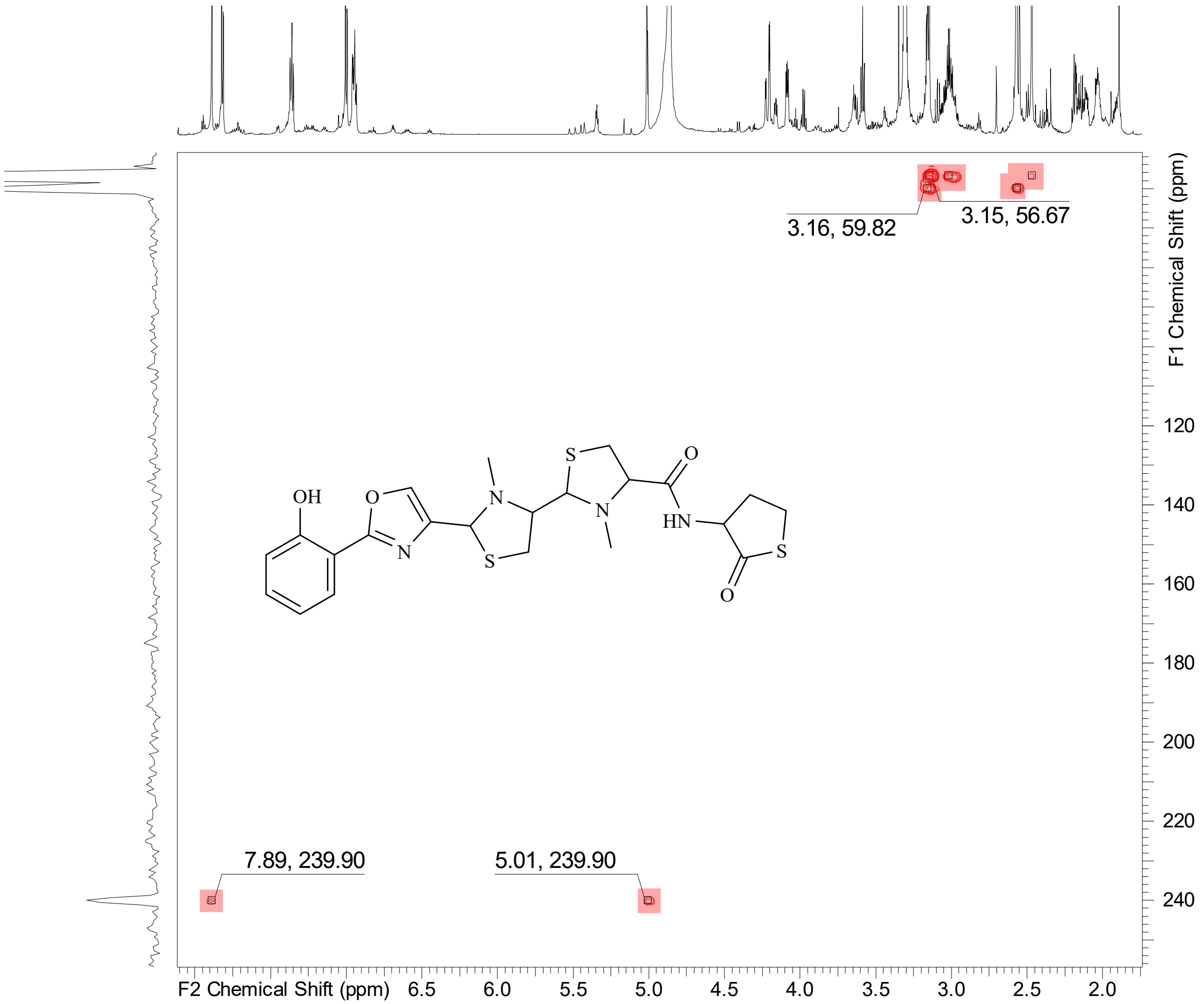

Figure S23 ^{1}H-^{15}N-HMBC spectrum of sorangibactin A2 (**2**) in methanol-*d*$_4$ at 500 MHz (^{1}H)/125 MHz (^{15}N).

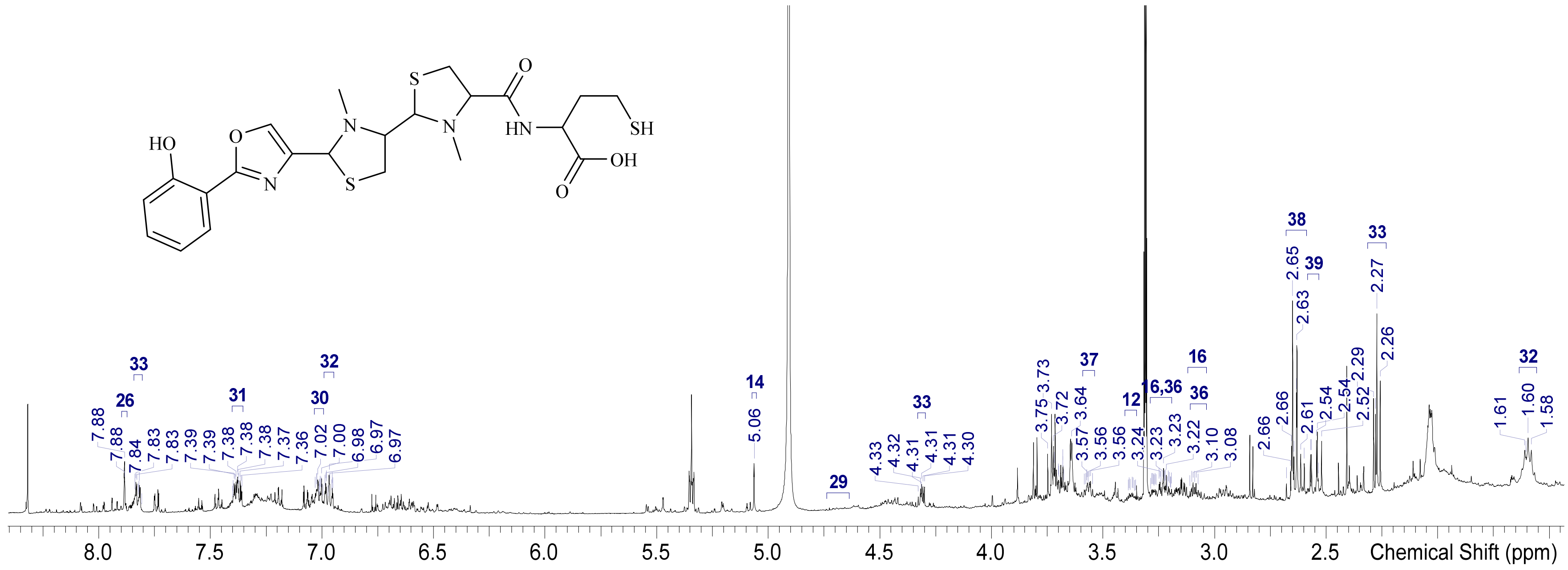

Figure S24 ^{1}H-spectrum of sorangibactin B (**3**) in methanol-d_4 at 500 MHz.

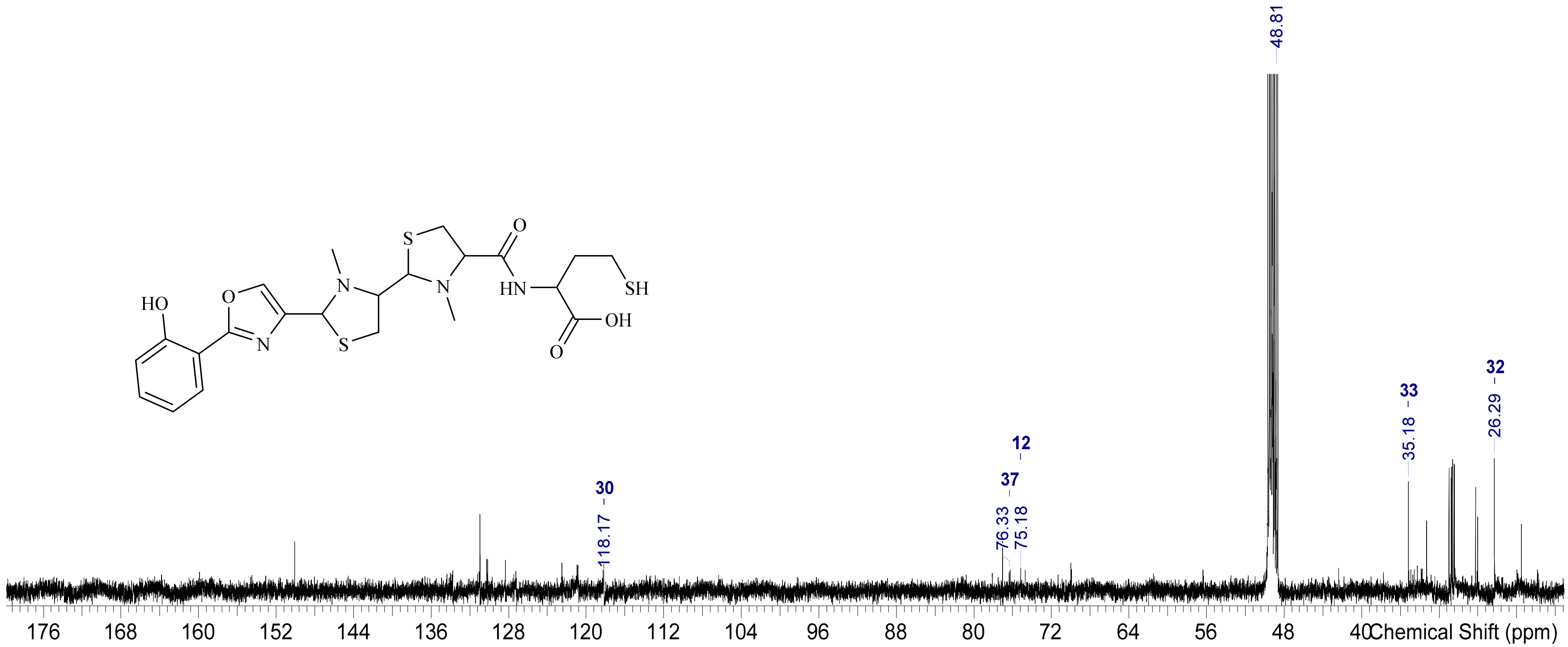

Figure S25 ^{13}C-spectrum of sorangibactin B (**3**) in methanol-d_4 at 125 MHz.

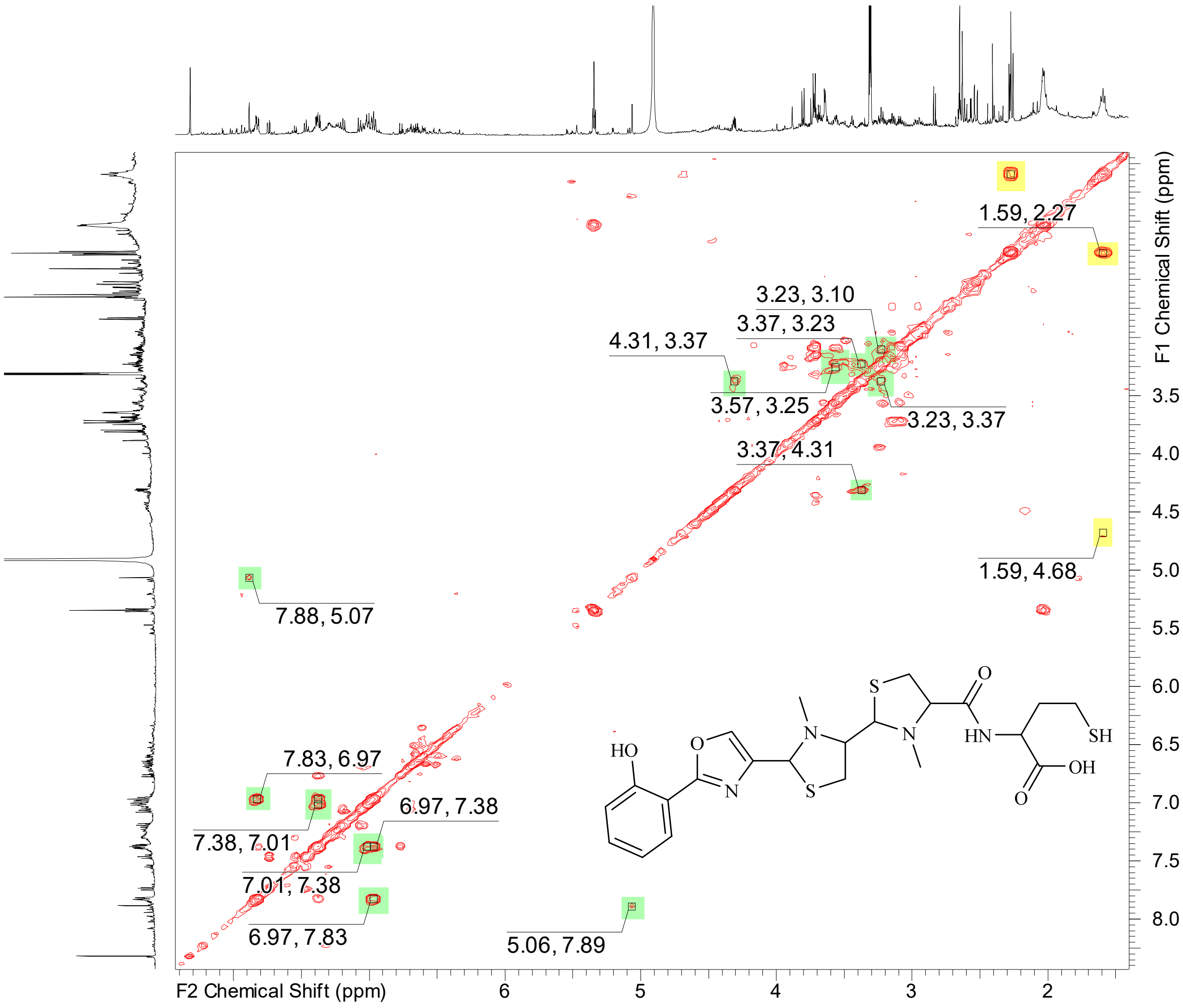

Figure S26 COSY spectrum of sorangibactin B (**3**) in methanol-d_4 at 500 MHz.

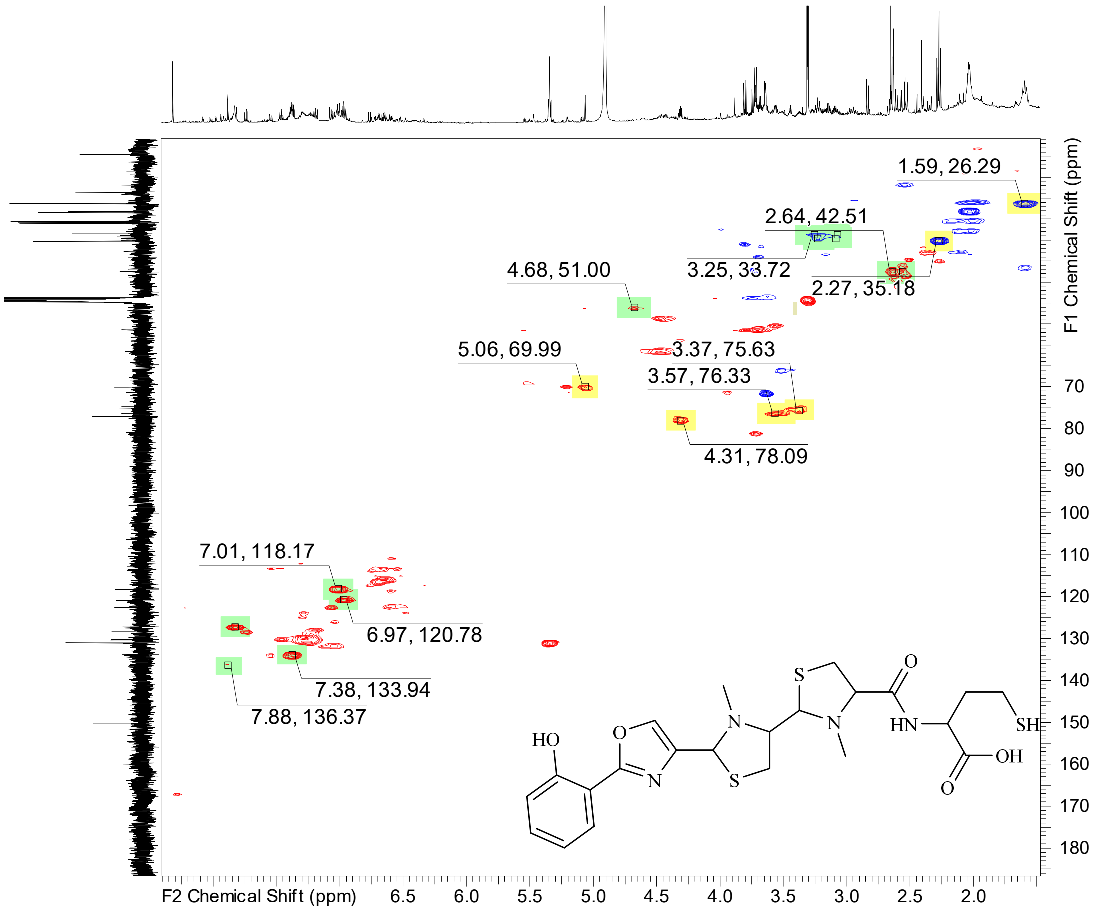

Figure S27 HSQC spectrum of sorangibactin B (**3**) in methanol-d_4 at 500 MHz (^{1}H)/125 MHz (^{13}C). Unpicked surrounding signals are most likely resulting from the presence of many different isomers in the same sample upon the acidic purification procedure required for the ring opened target compound of **3**.

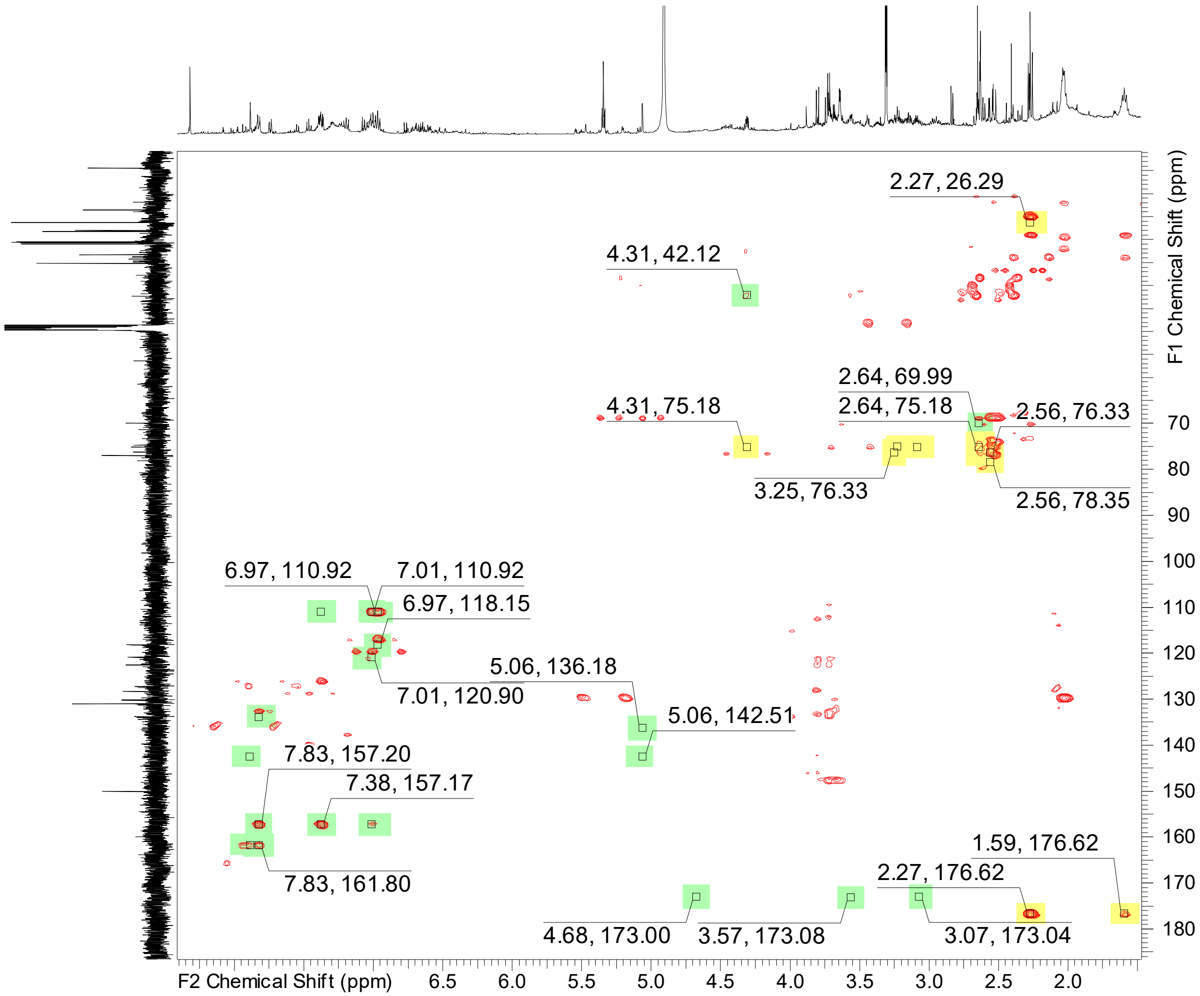

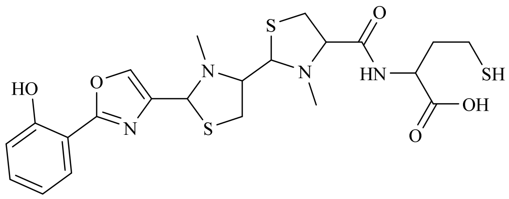

Figure S28 HMBC spectrum of sorangibactin B (**3**) in methanol-*d*4 at 500 MHz (^{1}H)/125 MHz (^{13}C).

4. References

1. Song, C.; Luan, J.; Li, R.; Jiang, C.; Hou, Y.; Cui, Q.; Cui, T.; Tan, L.; Ma, Z.; Tang, Y.-J.; Stewart, A. F.; Fu, J.; Zhang, Y.; Wang, H. RedEx: a method for seamless DNA insertion and deletion in large multimodular polyketide synthase gene clusters. *Nucleic Acids Res.* **2020,** *48* (22), e130. DOI: 10.1093/nar/gkaa956.

2. Perlova, O.; Fu, J.; Kuhlmann, S.; Krug, D.; Stewart, F.; Zhang, Y.; Müller, R. Reconstitution of myxothiazol biosynthetic gene cluster by Red/ET recombination and heterologous expression in *Myxococcus xanthus*. *Appl. Environ. Microbiol.* **2006,** *72* (12), 7485–7494. DOI: 10.1128/AEM.01503-06.

3. Magrini, V.; Creighton, C.; Youderian, P. Site-specific recombination of temperate *Myxococcus xanthus* phage Mx8: genetic elements required for integration. *J. Bacteriol.* **1999,** *181* (13), 4050–4061. DOI: 10.1128/JB.181.13.4050-4061.1999.

4. Jang, J.-P.; Kwon, M. C.; Nogawa, T.; Takahashi, S.; Osada, H.; Ahn, J. S.; Ko, S.-K.; Jang, J.-H. Thiolactomide: A New Homocysteine Thiolactone Derivative from *Streptomyces* sp. with Neuroprotective Activity. *J. Microbiol. Biotechnol.* **2021,** *31* (12), 1667–1671. DOI: 10.4014/jmb.2108.08015.

5. Mirdita, M.; Schütze, K.; Moriwaki, Y.; Heo, L.; Ovchinnikov, S.; Steinegger, M. ColabFold: making protein folding accessible to all. *Nat. Methods* **2022,** *19* (6), 679–682. DOI: 10.1038/s41592-022-01488-1.
